# Overexpression of oncogenic H-Ras in hTERT-immortalized and SV40-transformed human cells targets replicative and specialized DNA polymerases for depletion

**DOI:** 10.1371/journal.pone.0251188

**Published:** 2021-05-07

**Authors:** Wei-chung Tsao, Raquel Buj, Katherine M. Aird, Julia M. Sidorova, Kristin A. Eckert

**Affiliations:** 1 Department of Pathology, The Jake Gittlen Laboratories for Cancer Research, Penn State University College of Medicine, Hershey, Pennsylvania, United States of America; 2 Department of Cellular and Molecular Physiology, Penn State University College of Medicine, Hershey, Pennsylvania, United States of America; 3 Penn State Cancer Institute, Pennsylvania State University, Hershey, Pennsylvania, United States of America; 4 Department of Pathology, University of Washington, Seattle, Washington, United States of America; University of Minnesota Twin Cities, UNITED STATES

## Abstract

DNA polymerases play essential functions in replication fork progression and genome maintenance. DNA lesions and drug-induced replication stress result in up-regulation and re-localization of specialized DNA polymerases η and κ. Although oncogene activation significantly alters DNA replication dynamics, causing replication stress and genome instability, little is known about DNA polymerase expression and regulation in response to oncogene activation. Here, we investigated the consequences of mutant *H-RAS* ^G12V^ overexpression on the regulation of DNA polymerases in h-*TERT* immortalized and SV40-transformed human cells. Focusing on DNA polymerases associated with the replication fork, we demonstrate that DNA polymerases are depleted in a temporal manner in response to *H-RAS* ^G12V^ overexpression. The polymerases targeted for depletion, as cells display markers of senescence, include the Pol α catalytic subunit (*POLA1*), Pol δ catalytic and p68 subunits (*POLD1* and *POLD3*), Pol η, and Pol κ. Both transcriptional and post-transcriptional mechanisms mediate this response. Pol η (*POLH*) depletion is sufficient to induce a senescence-like growth arrest in human foreskin fibroblast BJ5a cells, and is associated with decreased Pol α expression. Using an SV-40 transformed cell model, we observed cell cycle checkpoint signaling differences in cells with H-Ras^G12V^-induced polymerase depletion, as compared to Pol η-deficient cells. Our findings contribute to our understanding of cellular events following oncogene activation and cellular transformation.

## Introduction

DNA replication is a critical phase of the cell cycle that must be tightly regulated to ensure accurate genome duplication. Failure to maintain DNA replication regulation leads to genome instability and ultimately tumorigenesis. Activating mutations in oncogenes alter DNA replication dynamics, promoting replication stress and genome instability [1, 2]. In turn, cells can undergo proliferative arrest known as oncogene-induced senescence, which acts as a tumorigenic barrier by preventing neoplastic transformation [1–4]. Replication stress is broadly defined as the slowing or stalling of the replication fork when the replisome encounters obstacles during DNA replication, and is associated with genome instability [5]. The replisome is a highly dynamic structure that incorporates multiple DNA polymerases, enzymes that are integral to all replication processes, including ongoing fork elongation, fork restart, and fork repair [6–10]. The regulation of various DNA polymerases to accomplish replication in response to exogenous, DNA damage-induced replication stress has been well studied [11, 12]. Critically, less is understood about the impact of oncogene-induced DNA damage on DNA polymerase regulation.

Significant evidence supports the hypothesis that the replication stress and DNA damage responses are induced during oncogene activation, prior to senescence [1, 13, 14]. Oncogenic Ras activation causes replicative stress, DNA damage, and senescence through a variety of mechanisms [1, 2, 15, 16]. Constitutively activated mutant *H-RAS* (hereafter referred to as Ras^G12V^) increases CDK2 activity and subsequent G1/S checkpoint abrogation, leading to increased origin firing, hyper-replication, and aberrant cell proliferation [1, 17]. Consequently, the prolonged presence of Ras^G12V^ activity is thought to cause replication stress through increased production of reactive oxygen species, replication-transcription machinery collisions, and depleted dNTP pools [16, 18–22].

The fidelity of genome replication is orchestrated by engaging multiple DNA polymerases [23]. Replicative polymerases delta (Pol δ) and epsilon (Pol ε) replicate the bulk of eukaryotic genomes under unstressed conditions, and are generally regarded as high fidelity [24]. Current models to explain resolution of stalled replication forks invoke specialized polymerases to perform DNA synthesis either at the fork, when replicative polymerases are inhibited [12], or post-replicative gap-filling synthesis behind the replication fork [25]. Specialized polymerases eta (Pol η) and kappa (Pol κ) maintain the integrity of genome duplication through DNA lesions, non-B DNA structures, and common fragile sites (CFS) [12, 26–30]. Correspondingly, replication stress caused by hydroxyurea, aphidicolin, and chemotherapeutic agents induce the up-regulation of Pol η, allowing cells to complete genome replication [31–33]. Recent research has shed some light on the DNA polymerases required to mitigate oncogenic stress. DNA Pol δ facilitates break-induced replication fork repair and cell cycle progression in cells overexpressing Cyclin E [34]. In normal human fibroblasts and cancer cells, Pol κ is important for the tolerance of Cyclin E/CDK2-induced DNA replication stress [35], while Pol η confers tolerance to Myc-induced replication stress in cancer cells [36].

Given their vital roles in maintaining genome stability, we sought to understand the regulation of DNA polymerases in response to oncogene activation, and we focused on cellular responses to mutant *H-RAS*. We discovered that several DNA polymerases are actively depleted in a temporal manner in response to Ras^G12V^ overexpression in human cells. Replicative polymerases appear to be regulated primarily at the transcriptional level, while specialized polymerases are regulated primarily at the post-transcriptional level. Finally, we show that, in SV-40 transformed cells, DNA polymerase depletion in response to Ras^G12V^ signaling is associated with slowed replication fork progression and enhanced Chk2 checkpoint activation. Our study shows that DNA polymerase expression is impacted by *H-RAS* activation, a fact that may contribute to oncogene-induced replication stress or genome instability.

## Materials and methods

A detailed list of Key Resources is provided in S1 Table.

### Cell culture and reagents

*hTERT*-immortalized BJ-5a human fibroblasts (CRL-4001^™^; ATCC) were cultured according to ATCC guidelines in 4:1 mixture of Dulbecco’s medium and Medium 1999 supplemented with 10% Hyclone^™^ FBS (GE Healthcare) and 50 μg/ml Gentimicin (Life Technologies). Experiments were performed between population doublings 10–35. SV40 transformed XPV and *POLH*-complemented cell lines (XP30RO) were a gift from Jean-Sebastian Hoffman (Cancer Research Center, Toulouse, France) and were cultured in Dulbecco’s medium, 10% FBS, and 50 μg/ml Gentimicin. Normal diploid IMR90 human fibroblasts (ATCC CCL-186) were cultured according to ATCC guidelines in low oxygen (2% O_2_) in DMEM with 10% FBS supplemented with L-glutamine, non-essential amino acids, sodium pyruvate, and sodium bicarbonate. Experiments with IMR90 were performed between population doublings 25–35. Cell lines were confirmed to be free of mycoplasma infection using MycoAlert^™^ Mycoplasma Detection Kit (Lonza).

### Retro and lentiviral packaging and infection

**Fig 1:**
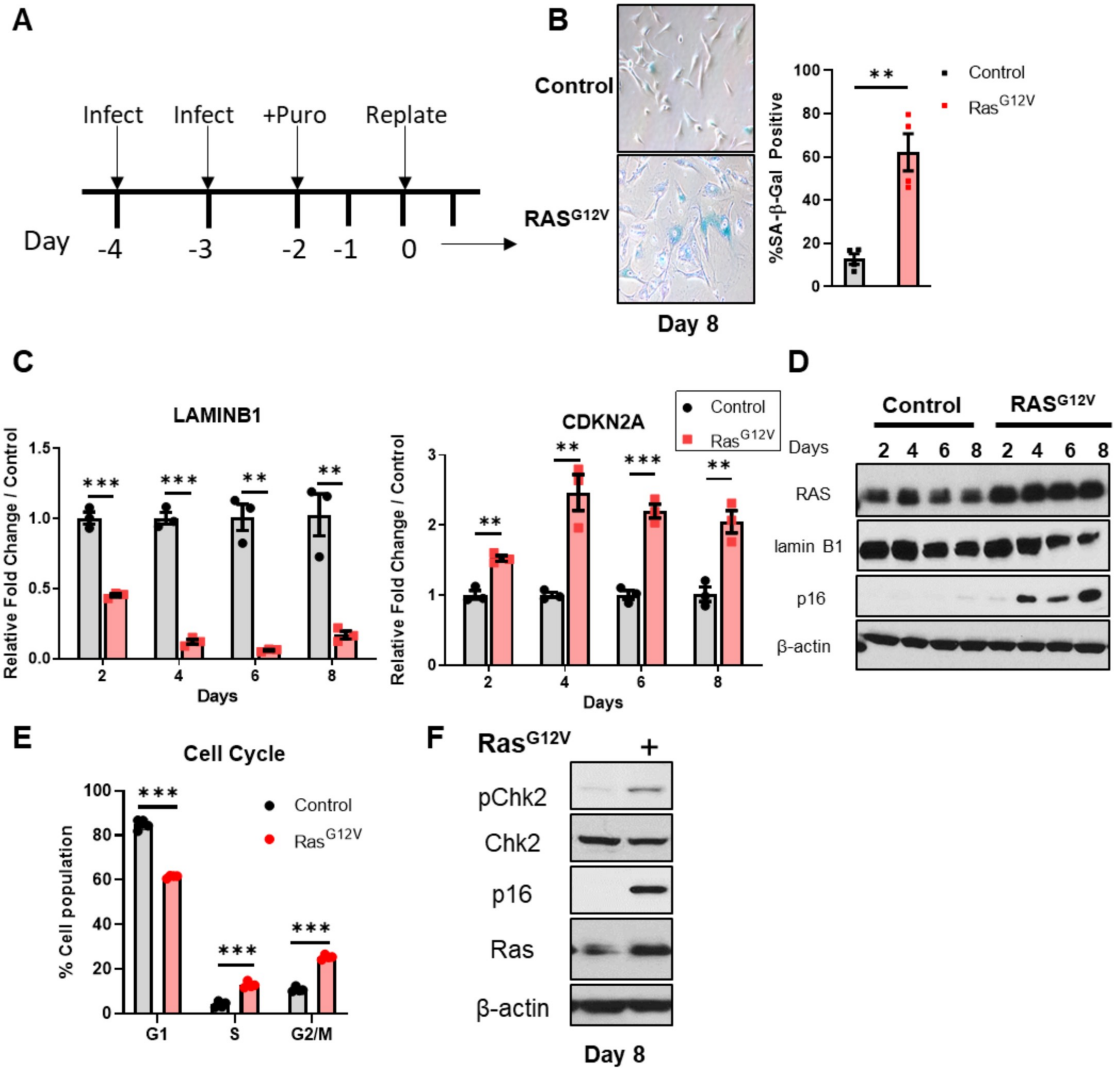
Ras^G12V^ overexpression in h*TERT*-BJ5a cells induces a senescent phenotype. (A) Schematic of the experimental infection and selection time course. (B) *hTERT* BJ5a human fibroblasts were infected with pBabe retrovirus empty vector (control) or encoding mutant Ras^G12V^. *Left panel*: SA- β-galactosidase staining of BJ5a cells (Day 8) with and without Ras^G12V^ OE. *Right panel*: Quantification of β-galactosidase staining (N = 3 technical replicates). Data represent mean +/- SEM. (C) mRNA expression of senescence markers *LMNB1* and *CDKN2A* after Ras^G12V^ OE. qRT-PCR was performed at the indicated timepoints following selection. Data represent mean +/- SEM of three biological replicates. (D) Corresponding immunoblot analysis of senescence markers LaminB1 and p16. One of three biological replicates is shown. (E) Cell cycle analyses of control and Ras^G12V^ OE cells on Day 8. Data represent mean +/- SD of three biological replicates. (F) Checkpoint activation after Ras OE. Immunoblot analyses for Chk2 Thr38 phosphorylation was performed on Day 8. One of three biological replicates is shown.

Retrovirus production from pBabe vectors was performed using 293FT phoenix cells and human cell transduction was performed using the BBS/calcium chloride method (15). Lentivirus was packaged using the ViraPowerKit (Thermofisher) following the manufacturer’s instructions with modifications. Briefly, pLKO.1 Lentiviral constructs (Addgene) were transfected into 293FT cells using 35 μg polyethylenimine per transfection (Alfa Aesar). After 48 hours of incubation, virus-containing media was collected. The experimental timeline of human cell infections and selection is outlined in Fig 1A. Cells were infected with viruses containing pBabe vector-only (control) or pBabe encoding HRas^G12V^, followed by a second round of infection 24 hours later. 24 hours after the second infection, the cells were replated, and selected with puromycin 6 hours after seeding (2 μg/μl for *hTERT* BJ5a; 1 μg/μl SV40 cell lines). For dual Ras^G12V^ and shRNA lentiviral infections, BJ5a cells were first infected with viruses containing pBabe vector-only or HRas^G12V^ vectors. After 24 hours, a second round of infections was performed simultaneously with viruses containing both pBabe vectors and pLKO.1 vectors as described above. Cells were replated and selected with 4 μg/ml puromycin for 2 days, followed by reseeding for subculture and assays. For infections with inhibitor treatments, infected cells were plated into 10 cm^2^ plates the day before the indicated timepoints. After 24 hours, cells were treated with proteasome inhibitor MG132 (Sigma) for 4 hours, followed by protein isolation (described below).

### Gene expression analysis

Experiments were performed according to MIQE guidelines with at least three technical replicates for all cell lines and three biological replicates for BJ5a cells. Total RNA was extracted using RNAeasy Kit (Qiagen), assessed for quality using the 2200 TapeStation (Agilent), and 600 ng-1 ug of samples with RIN>9 were converted to cDNA using qScript cDNA Synthesis Kit (Quanta Bioscences). qPCR was performed according to manufacturer guidelines with 20 ng of cDNA, 1X Taqman target and control probes (Life Technologies), and PerfeCTa^Ⓡ^ Fast Mix^Ⓡ^ II, Low Rox (Quanta Biosciences). The reactions were analyzed using Agilent QuantStudio 7 Flex.

### Immunoblot analysis and quantification

Whole cell extracts were collected by lysing cells with RIPA buffer (Santa Cruz Biotechnology) supplemented with Halt protease and phosphatase inhibitors (Life Technologies) and PMSF (Santa Cruz). Extracts concentrations were determined using DC^™^ Protein Assay (Bio-Rad). Sample preps were prepared using 4X LDS Sample buffer and 10X Reducing Agent (Life Technologies) and incubated at 70°C for 10 min. Samples were loaded into pre-casted NuPage gels (Life Technologies). Gels were electrophoresed in 1X MOPS buffer, transferred onto 0.2um Amersham^™^ Hybond^™^ PVDF membranes (GE Healthcare). After transfer, efficiency was visualized by Ponceau S staining and blocked with 5% non-fat milk or BSA in TBS containing 0.1% Tween (TBST) before incubating with primary antibody overnight. Membranes were washed 5 times with TBST for at least 10min and were incubated with mouse or rabbit secondary antibodies at 1:20,000 dilution for 1 hour at room temperature. After washing with TBST for 5 times, blots were visualized with chemiluminescence reagents Amersham^™^ ECL^™^ Prime Western Blotting Detection Reagents (GE Healthcare) or Pierce^™^ ECL Western blotting substrate (Thermofisher). Bands were quantified using ImageJ (NIH) [37]. Briefly, relative intensity was calculated for individual bands per blot. Adjusted intensities were calculated by normalizing relative intensity of each band to its respective β-actin control.

### Beta-galactosidase staining

Senescence-associated (SA)-β-galactosidase staining was performed as previously described [38]. Briefly, cells were fixed with 2% formaldehyde/0.2% glutaraldehyde in PBS for 10 min. After washing with PBS, cells were stained at 37°C in a non-CO_2_ incubator with the staining solution (40 mM Na_2_HPO_4_ pH 5.7, 150 mM NaCl, 2 mM MgCl_2_, 5 mM K_3_Fe(CN)_6_, 5 mM K_4_Fe(CN)_6_, 1 mg/ml X-gal). After 16 hours, wells were washed with RO water and images were acquired using an inverted microscope (Nikon Eclipse Ti) with a 20X/0.45 objective and a camera (Nikon DS-Fi3).

### Immunofluorescence for EdU incorporation

Cells were plated on 22x22 mm glass coverslips the day before indicated time point (Fisher Scientific). Cells were pulsed with 20 μM EdU(5-ethynyl-2’deoxyuridine Lumiprobe) for 1 hour and fixed with 4% paraformaldehyde (Sigma) for 10 min. Cells were washed with PBS and permeabilized with 0.3% Triton-X for 15 min. Reaction mixes were freshly made with 20 uM FAM-azide (Lumiprobe B5130), 4 mM copper sulfate pentahydrate (Sigma), 20 mg/ml ascorbic acid (Sigma) in PBS. Coverslips were labeled with 40 μl reaction mix for 30 min at room temperature. Coverslips were washed twice with PBS and mounted with antifade mounting medium VECTASHIELD^Ⓡ^ with DAPI (Vectorlabs). Images were acquired at room temperature using Zeiss AXIO Microscope Imager.M2 and Apotome.2 apparatus with a 64X oil objective and the Zen Pro software. Representative pictures were obtained using Z-stacks and maximum intensity projections.

### Cell cycle analysis

After infection, cells were fixed in 70% ethanol, washed, resuspended with 1% BSA in PBS, and placed in -20°C for at least overnight until further processing. For standard cell cycle analysis, cells were resuspended with 0.1% Triton-X, 200 μg/ml RNAase, and 40μg/ml propidium iodide. For EdU cell cycle analysis, cells were pulsed with 20 μM EdU for 1 hour. Cells were harvested using trypsinization, fixed with 4% paraformaldehyde for 15 min, and permeabilized with 0.1% saponin (47036-50G-F Sigma) and 1% BSA in PBS (Saponin-BSA buffer) for 15 min. Cells were washed Saponin-BSA buffer and incubated with Click reaction buffer containing 20 μM Sulfo-cyanin5 azide (Lumiprobe B3330), 4mM copper sulfate pentahydrate, and 20 mg/ml ascorbic acide in PBS for 30min. Cells were washed with Saponin-BSA buffer twice and incubated with 200 μg/ml RNAse and 40 μg/ml propidium iodide at 37°C for 15 min. Samples were analyzed on BD FACSCanto with FlowJo software. Cells with >4C DNA content were excluded from the analyses.

### Clonogenic survival

After retroviral infection, cell populations were selected with puromycin for 2 days, followed by seeding at 1000 cells per well (in triplicate) using 6-well plates. Media was replaced every 2–3 days. After 10–14 days, colonies were fixed with 100% methanol for 10 min and stained with crystal violet solution for 15 min. Wells were destained using 10% acetic acid and the intensities of crystal violet staining were quantified using 590 nm absorbance with a spectrophotometer. Raw values were normalized to a control well with no cells. Survival percentages were calculated as the mean normalized staining values from Ras^G12V^-infected cells divided by the mean normalized staining values from control-infected cells.

### Microfluidics-assisted replication track analysis (maRTA)

Fiber combing analyses were performed as previously described [39, 40]. Briefly, the day before the indicated timepoint, cells were plated in 60 cm plates at 60% confluency overnight. Cells were labelled with 50 μM IdU for 30 min, washed 3 times with PBS, and labelled with 250 μM CldU for 30 min. Cells were trypsinized and washed with agarose insert buffer (10 mM Tris7.5, 20 mM NaCl, 50 mM EDTA in water). After spin down, cells were resuspended in agarose insert buffer and mixed 1:1 with 2% low-melting agarose (Bio-Rad). Gel inserts were solidified at 4°C overnight and were stored in agarose insert buffer until analysis. Microscopy of stretched DNAs was performed on the Zeiss Axiovert microscope with a 40x objective, and images were captured with the Zeiss AxioCam HRm camera. Fluorochromes were Alexa594 for CldU and Alexa488 for IdU. Lengths of tracks were measured manually in raw merged images using Zeiss AxioVision software, as well as automatically, using an open source software FiberQ [41] with concordant results. Percentages of ongoing (IdU-CldU) or terminated (IdU only) forks, and origin firing events were derived from FiberQ outputs. Statistical significance for track lengths was calculated using Kruskal-Wallis tests followed by pairwise Wilcoxon tests to derive p values adjusted for multiple comparisons. For origin firing percentages, statistical significance was determined in one-way ANOVA with post-hoc analysis with Tukey’s test to derive p values adjusted for multiple comparisons. Analyses were done in R Version 3.6.3.

### Statistics

Unless stated otherwise, graphical representation and statistical analyses were done using GraphPad Prism v8.4.1. Unless stated otherwise in the figure legends, all statistical analyses were performed using unpaired Student’s t-test, two-tailed. *p<0.05, **p<0.01, ***p<0.005, ****p<0.001, n.s., not significant.

## Results

### Overexpression of oncogenic H-Ras^G12V^ induces differential depletion of DNA polymerases in nontumorigenic human cells

We aimed to elucidate DNA polymerase expression in response to oncogene-induced replication stress, using an established experimental model of *H-RAS* ^G12V^ overexpression (hereafter referred to as Ras^G12V^ OE) [1, 42] and *hTERT*-immortalized BJ5a human fibroblasts, which do not undergo the replicative senescence of primary fibroblasts [43, 44]. In response to oncogene activation, these cells activate a senescence program, followed by some cells escaping senescence 2–3 weeks after oncogene overexpression [45]. We infected *hTERT*-BJ5a with a constitutively active, Ras^G12V^ expressing retroviral vector or an empty vector control, followed by puromycin selection for stable transductants (Fig 1A). As expected, we observed an induction of several senescent phenotypes over the course of several days in Ras^G12V^ OE cells, including increased senescence-associated (SA)-β-galactosidase staining, decreased laminB1 expression and increased CDKN2A/p16 expression, compared to control-infected cells (Fig 1B–1D). After 8 days of Ras^G12V^ OE, we measured significantly altered cell cycle distributions and increased CHK2-Thr38 phosphorylation (Fig 1E and 1F). Together, these data show the expected onset of senescent phenotypes and increased DNA damage checkpoint response after Ras^G12V^ OE.

**Fig 2:**
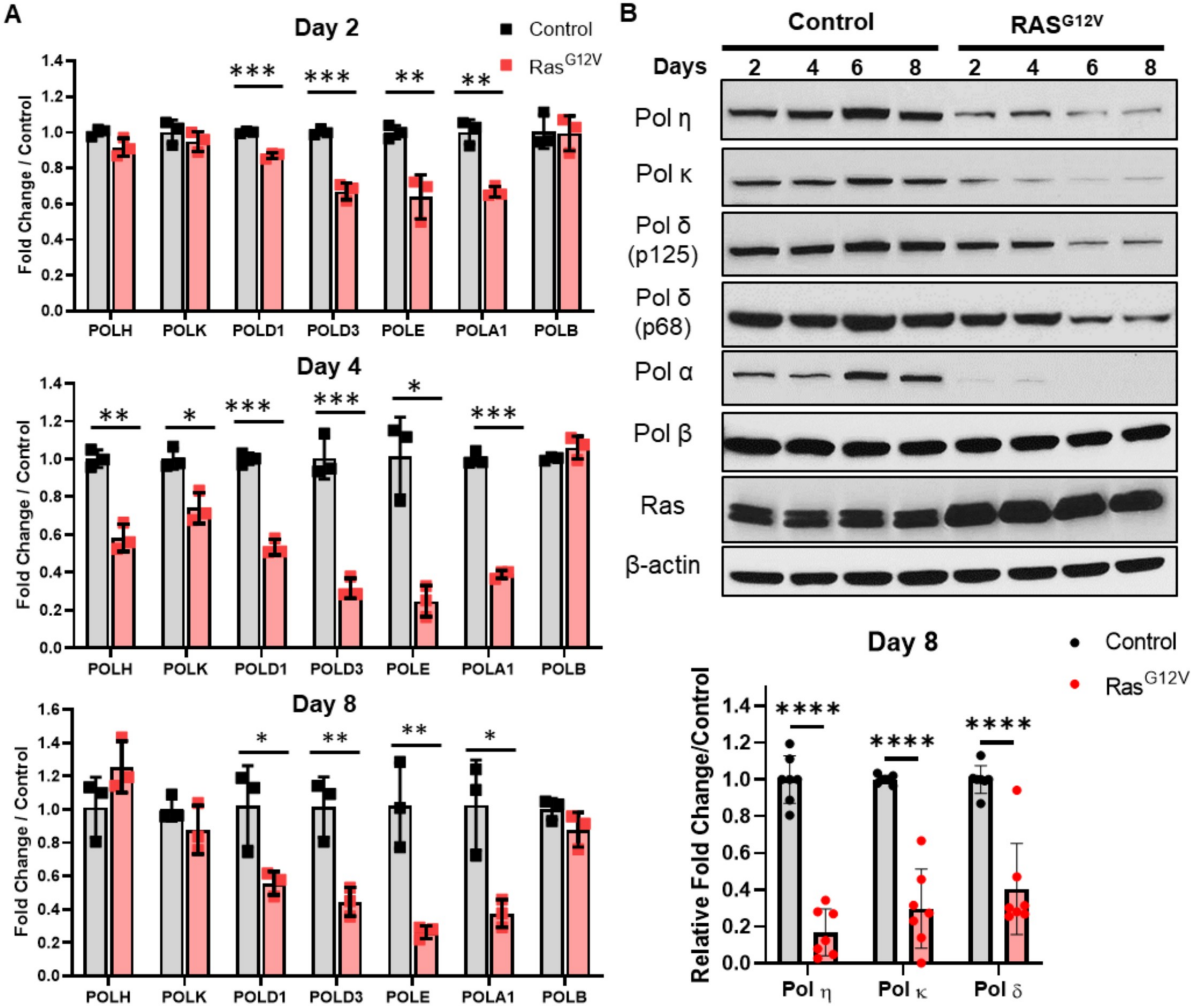
Overexpression of oncogenic Ras^G12V^ induces downregulation of replication fork-associated DNA polymerases. (A). *hTERT* BJ5a human fibroblasts were infected with pBabe retrovirus empty vector (control; gray bars) or encoding mutant Ras^G12V^ (red bars). POL gene expression was determined using qRT-PCR at the indicated timepoints after selection. Data represent mean +/- SEM of three biological replicates. (B). *Top panel*-Immunoblot analyses of representative control and Ras^G12V^ -infected cell populations at indicated timepoints. One of three biological replicates is shown. *Bottom panel-* Quantification of DNA polymerase protein levels 8 days after control or Ras^G12V^ transduction. Data represent mean +/- SD of seven biological replicates.

Next, we measured the expression of several DNA polymerase genes in Ras^G12V^ and control infected cells, as a function of days following oncogene overexpression and the onset of senescent phenotypes. Strikingly, we observed significant downregulation of several replicative polymerase genes, including *POLA1*, *POLD1*, *POLD3*, and *POLE*, after Ras^G12 V^ OE as early as day 2 post selection, and sustained through day 8 (Fig 2A). In contrast, we found only transient and slight downregulation of specialized polymerase genes *POLH* and *POLK* at Day 4, while the DNA repair polymerase *POLB* gene expression was not affected at any time point measured.

DNA polymerase protein levels were also significantly and differentially impacted by Ras^G12V^ OE (Fig 2B). The catalytic subunit of replicative Pol α was depleted as early as day 2. Moreover, production of Pols η, κ, and both catalytic (p125; *POLD1* gene) and accessory (p68; *POLD3* gene) Pol δ subunits was significantly depleted after 8 days. However, similar to its mRNA expression, Pol β protein levels are not altered after Ras^G12V^ OE. Taken together, these results show that DNA polymerases are differentially regulated at the transcript and protein levels in response to oncogenic Ras^G12V^ overexpression.

### Ras^G12V^ induced depletion of DNA polymerases is dependent on the proteasomal degradation pathway

**Fig 3:**
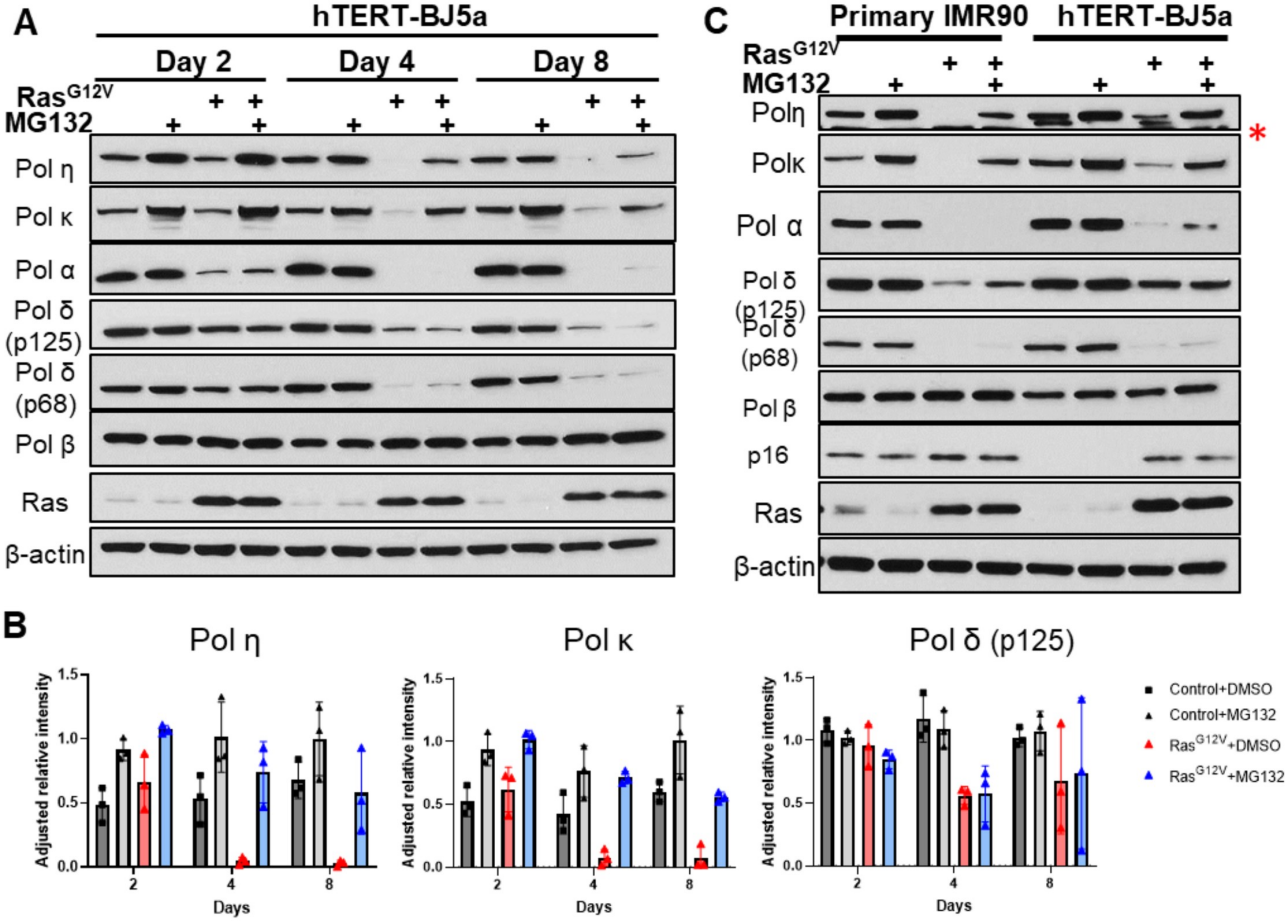
DNA polymerases η and κ levels are regulated by the proteasome degradation pathway. (A) Immunoblot analyses of polymerase levels at indicated days after transduction and selection with control or Ras^G12V^ vectors. *h-TERT* BJ5a cells were either treated with DMSO or MG132 (10μM) for 4 hours prior to harvesting. Immunoblot analysis represent one of three biological replicates (see S1 Fig for additional replicates). (B) Relative polymerase levels in BJ5a cells. Adjusted relative intensity values were calculated using ImageJ; data are from three biological replicates. (C) Side-by-side comparison of primary fibroblast IMR90 and BJ5a cells infected with control or Ras^G12V^ at day 8. Cells were treated with DMSO or MG132 (10 μM) for 4 hours prior to harvest. The BJ5a results shown here are an independent biological replicate of Fig 3A. *Asterisk indicates a non-specific band.

We tested whether the depletion of DNA polymerase proteins after Ras^G12V^ OE is due to proteasomal degradation. For each time point after Ras^G12V^ OE, *hTERT*-BJ5a cells were treated with MG132, a 26S proteasome inhibitor, for 4 hours immediately prior to harvesting cell lysates for analysis. Remarkably, we observed a robust rescue of specialized Pols η and κ at multiple timepoints (Fig 3A and 3B; S1 Fig) in treated cells expressing Ras^G12V^. Notably, the levels of rescue diminish over time after Ras^G12V^ expression. Pols η and κ protein levels are also slightly increased after MG132 treatment of control cells, consistent with previous reports [46, 47]. In contrast, we observed little to no rescue of Pols α (catalytic subunit) or δ (p125 or p68 subunits) in BJ5a cells (Fig 3A and 3B). This result is consistent with replicative polymerase regulation in response to Ras^G12V^ OE occurring primarily at the transcriptional level (Fig 2A). To examine the generality of this response, we repeated this experiment using IMR90 primary fibroblasts. Pols η, κ, and δ are again depleted after Ras^G12V^ OE, and this degradation can be rescued, at least in part, by MG132 treatment (Fig 3C). Together, these data are consistent with DNA Pols η and κ levels being primarily regulated post-transcriptionally and, in part, through the proteasome degradation pathway, under Ras^G12V^ signaling.

### Correlation of senescence-associated p16^INK4A^ and DNA polymerase depletion in response to Ras^G12V^ overexpression

**Fig 4:**
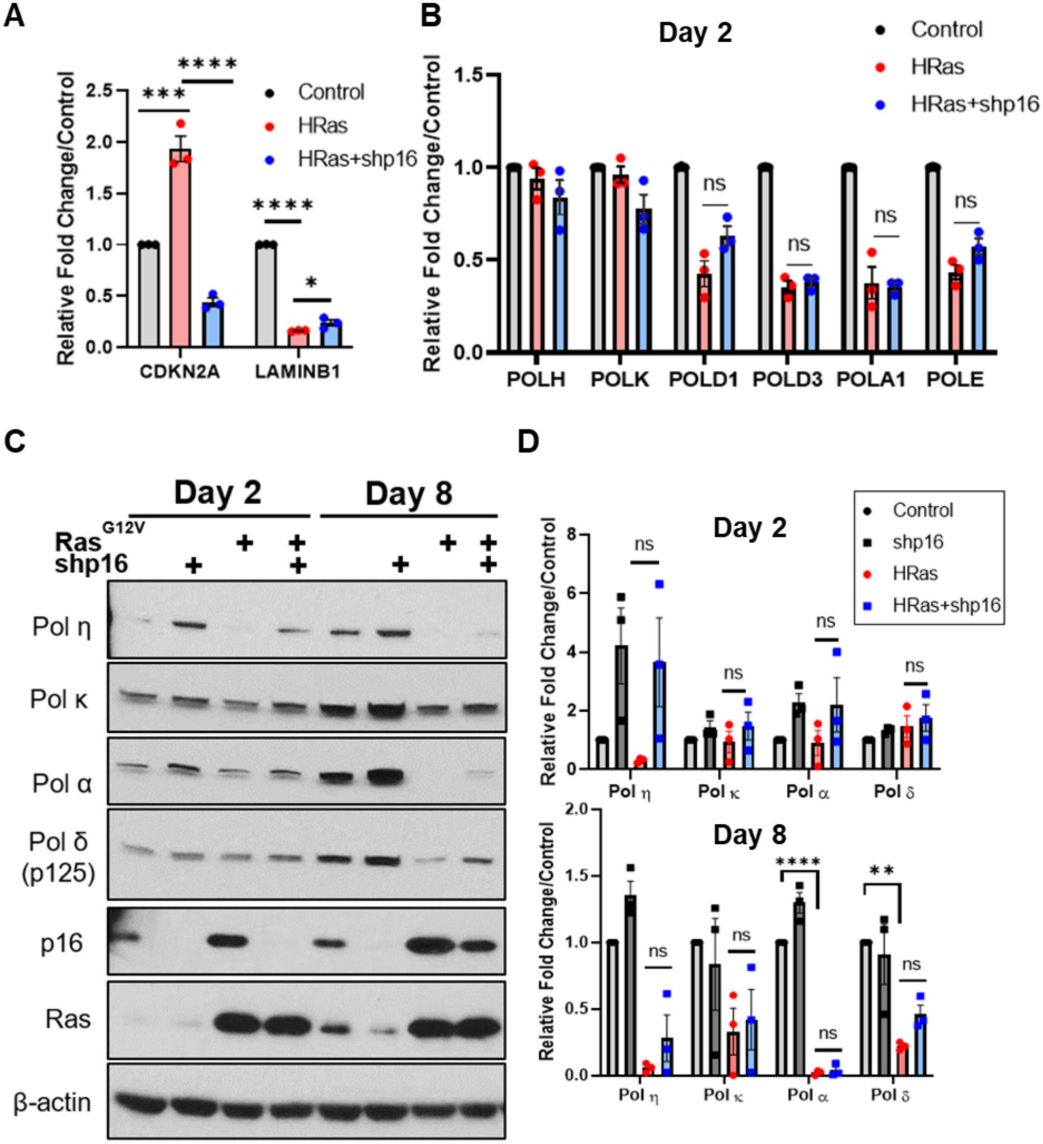
Relationship of p16 to DNA polymerase expression in response to oncogenic Ras^G12V^ signaling. *hTERT*-BJ5a cells were infected with lentivirus expressing short hairpin RNAs (shRNAs) targeting *CDKN2A*. Scrambled shRNA was used as control. (A) mRNA expression analysis of senescence markers on Day 2 as determined using qRT-PCR. (B) mRNA analyses of DNA polymerase gene expression on Day 2 as determined using qRT-PCR. Data represent mean +/- SEM from three biological replicates. Statistical analyses were performed using One-way ANOVA with Tukey’s post-hoc. n.s., not significant. *p<0.05, ***p<0.005, ****p<0.0001. (C) Immunoblot analyses of control or Ras^G12V^ BJ5a cells, with and without p16 knockdown. Data are representative of three independent replicates. (D) Quantification of control or Ras^G12V^ BJ5a cells immunoblot analyses, with and without p16 knockdown. Statistical analyses were performed using One-way ANOVA with Tukey’s post-hoc. **p<0.005, ****p<0.0001, n.s., not significant.

The tumor suppressor gene *CDKN2A* (p16^INK4A^) is a major regulator of senescence [48–50]. To ascertain whether p16^INK4A^ plays a role in the observed DNA polymerase depletion, we used an shRNA approach to knockdown *CDKN2A* (p16 ^INK4A^) expression after Ras^G12V^ infection (Fig 4A). At the transcript level, we observed no significant change in the expression of the polymerase genes examined after *CDKN2A* knockdown (Fig 4B). At the protein level, p16^INK4A^ levels were the lowest in Ras^G12V^ OE + sh*CDKN2A* cells at day 2. After 8 days of Ras^G12V^ OE, p16 levels are elevated even in the presence of lentiviral sh*CDKN2A* (Fig 4C). While we observed some increase in Pols α, δ (p125), and κ levels with p16 knockdown, these effects were variable and not statistically significant (Fig 4D). However, we did observe a pattern that Pol η protein expression may be inversely correlated with the presence of p16^INK4A^.

### Pol η depletion is sufficient to induce a senescence-like growth arrest

**Fig 5:**
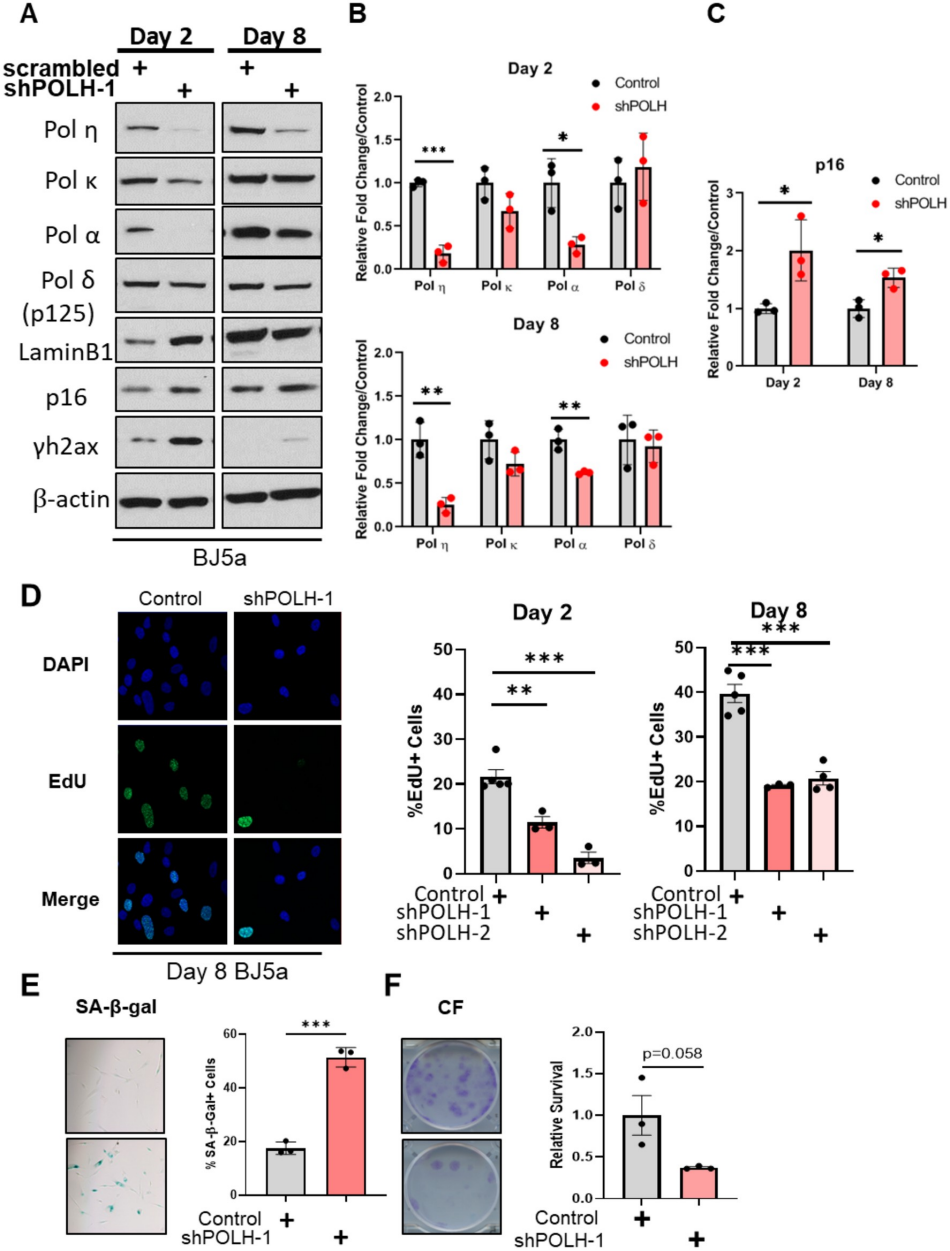
DNA polymerase η depletion is sufficient to induce a senescence-like growth arrest. (A). *hTERT*-BJ5a cells were infected with lentivirus expressing shRNA targeting *POLH*. Scrambled shRNA was used as control. Immunoblot analyses of indicated proteins at days 2 and 8 after lentivirus infection is representative of three biological replicates. (B). Quantification of immunoblot analyses of BJ5a cells infected with *POLH* or scrambled shRNA lentivirus at days 2 and 8. Data represent mean +/- SD of three biological replicates. (C). Quantification of p16 expression in BJ5a cells infected with *POLH* or scrambled shRNA lentivirus at days 2 and 8. Data represent mean +/- SD of three biological replicates. (D). Quantification of EdU positive cells (by immunofluorescence) of either two separate shRNA clones or scrambled shRNA at indicated days. Data represent mean +/- SEM for at least three biological replicates with two technical replicates. (E). Quantification of SA-β-gal activity at Day 8. Data represent mean +/- SEM for three biological replicates. (F). Quantification of clonogenic survival (crystal violet staining intensity) at Day 14. Data represent mean +/- SEM for three biological replicates.

Both Pol η and Pol κ play important roles in genome duplication, DNA damage, and the replication stress response [11, 12, 29]. We and others have shown that Pol η mRNA and protein levels are increased in tumor cells in response to exogenous sources of replication stress [31–33]. Therefore, the HRas^G12V^-induced depletion of Pols η and κ in cells undergoing senescence was surprising. However, previous reports have hinted that Pol η may play a role in regulating senescence. Pol η knockout mice display metabolic abnormalities and increased senescence-associated phenotypes specifically in adipocytes [51]. Pol η also could suppress senescence because it participates in the alternative lengthening of telomeres (ALT) pathway [52]. Therefore, we tested directly whether loss of Pol η expression is sufficient to induce senescence. To do so, we transduced *hTERT*-BJ5a cells with lentiviral *POLH* shRNAs. Strikingly, we found a significant reduction of Pol α protein expression (Fig 5A and 5B) concomitant with *POLH* knockdown. *POLH* knockdown cells also exhibited increased p16 expression, compared to controls (Fig 5C). *POLH* depletion reduced the number of EdU positive cells on both days 2 and 8 (Fig 5D). *POLH* knockdown also increased SA-β-galactosidase staining and decreased clonogenic survival (Fig 5E and 5F). Taken together, these results suggest that depletion of Pol η is associated with decreased Pol α and increased p16 expression and is sufficient to induce a senescence-like growth arrest.

### DNA polymerase degradation in response to Ras^G12V^ in SV40 transformed cells is associated with altered replication fork dynamics

**Fig 6:**
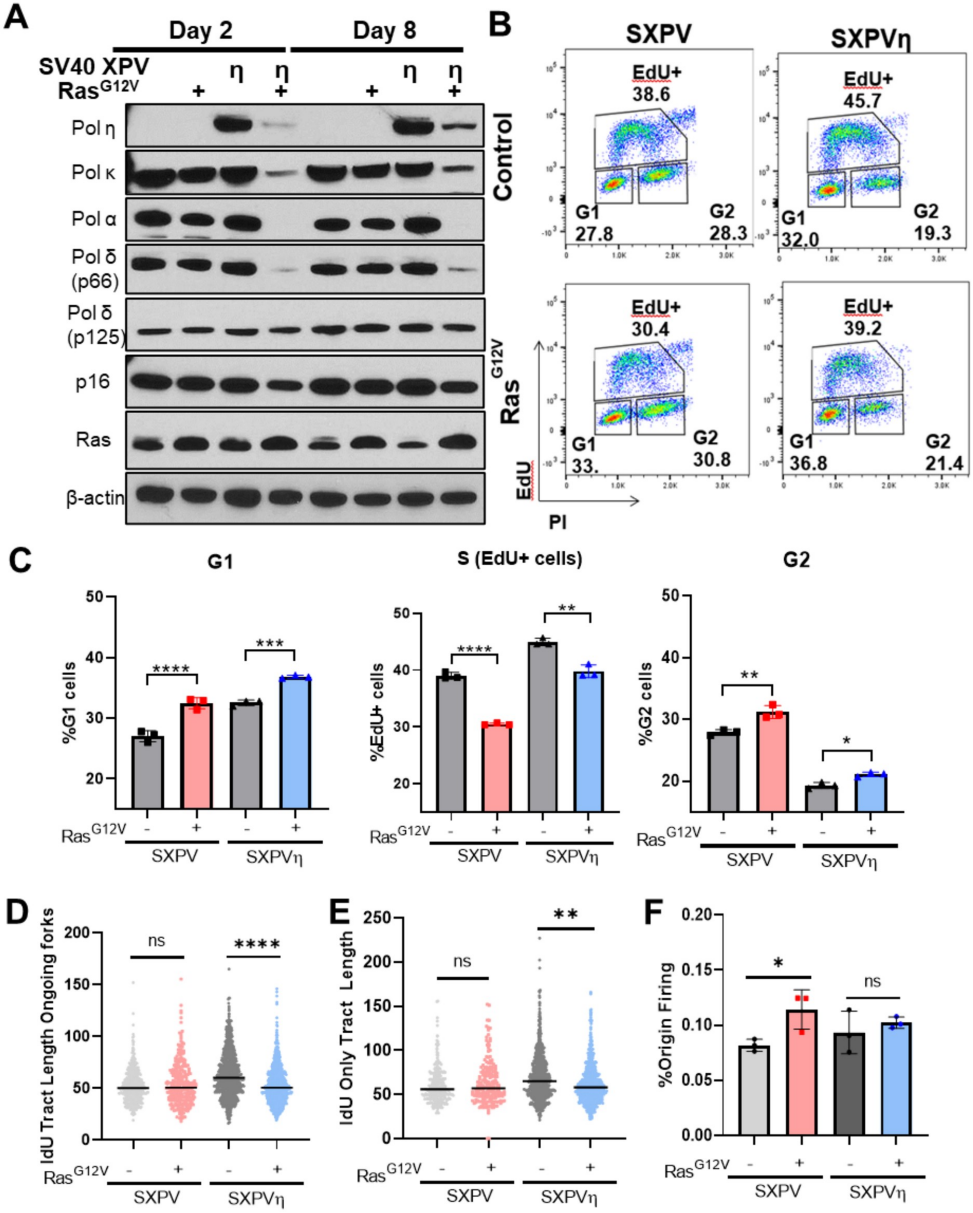
Differential oncogenic Ras^G12V^-induced DNA polymerase depletion is associated with altered replication fork dynamics. (A). Immunoblot analysis of SV40-transformed XPV cells (SXPV) and *POLH*-complemented SV40-transformed XPV cells (SXPVη), infected with control or Ras^G12V^ at Days 2 and 8. One of four biological replicates is shown. (B). Representative cell cycle analyses of SXPV/SXPVη cell populations infected with control vector or Ras^G12V^ at Day 8. (C). Quantification of cell populations (B) from three biological replicates. (D). DNA fiber combing assay for ongoing (IdU-CldU) forks. Data represent IdU lengths of IdU-CldU tracts from two biological replicates. Statistical analysis was performed using Wilcoxon rank sum test with Holm’s post-hoc test. ****p<0.0001. n.s., not significant. (E). DNA fiber combing assay for terminated (IdU only) forks. Data represent IdU tract lengths from IdU only fiber of two biological replicates. Statistical analysis was performed using Wilcoxon rank sum test with Holm’s post-hoc test. **p<0.01. n.s., not significant. (F). DNA fiber combing assay for origin firing. Percent origin firing was calculated using CldU only and CldU-IdU-CldU tracts / total tracts. Origin firing data is from three biological replicates.

DNA polymerase levels can be altered in human tumors (8); specifically, *POLH* is upregulated or amplified in melanoma, esophageal and ovarian cancer [28, 32, 53, 54], which contrasts with the HRas^G12V^-induced depletion we measured above in nontumorigenic cells. Therefore, we next asked whether the degradation of DNA polymerases after oncogene activation persists in transformed cells. To mimic a tumor-like model, we used immortalized, SV40 virus-transformed XPV30RO fibroblasts (SXPV) [55], in which the p53, Rb, and other cancer-associated signaling pathways are disrupted. To mimic the increased expression of Pol η observed in tumors, we utilized genetically related SV40 transformed, *POLH*-complemented XPV30RO cells (SXPVη), which overexpress Pol η [56]. Similar to our observation using *hTERT* and primary cell lines, Ras^G12V^ OE in SXPVη cells led to a rapid depletion of Pols α, η, κ and δ (p68 subunit) as early as day 2 and sustained through Day 8; however, depletion of the Pol δ catalytic p125 subunit was not observed (Fig 6A). Moreover, the depletion of exogenous Pol η in SXPVη (*POLH*-complemented) cells is further evidence that the regulation of Pol η is post-transcriptional.

In striking contrast, we observed no depletion of any DNA polymerase analyzed in genetically related SXPV (Pol η-deficient) cells after Ras^G12V^ OE (Fig 6A). Having this pair of cell lines with distinct responses afforded us the opportunity to investigate the consequences of Ras-induced polymerase degradation on cellular processes. Replicative Pols α and δ are required for the bulk of on-going genome replication [10]. Therefore, we asked what impact the depletion of Pol α DNA polymerase catalytic subunit (p180) and Pol δ accessory subunit (p68) has on cell cycle progression and DNA replication. In both SXPV and SXPVη cells, Ras^G12V^ OE resulted in similar cell cycle changes (Fig 6B), relative to the corresponding control infected cells, with increased populations of cells in G1and G2 phases and decreased populations of cells in S phase (Fig 6C). However, even after 8 days of Ras^G12V^ OE, both SXPV and SXPVη populations retain ~30–40% of EdU+, S phase cells, despite the differential DNA polymerase expression. Typically, cells undergoing replication stress display slower fork progression, which can be compensated by increased origin firing [2]. Using DNA fiber analyses, we determined that the depletion of DNA polymerases in SXPVη cells after Ras^G12V^ OE led to a significant decrease in the length of ongoing and terminated forks, as expected for fork slowing (Fig 6D and 6E). However, we measured no increase in origin firing (Fig 6F), possibly due to low Pol α levels, a critical enzyme required for origin activation. In contrast, in Ras^G12V^-infected SXPV cells where DNA polymerases α and others are present, we observed no changes in fork progression (Fig 6D and 6E). Instead, Ras^G12V^-infected SXPV cells displayed increased origin firing, compared to control infected cells (Fig 6F). Together, these data suggest that depletion of DNA polymerases in SXPVη cells after 8 days of Ras^G12V^ leads to reduced replication fork elongation but no significant changes in origin activation.

### DNA polymerase degradation in response to Ras^G12V^ in SV40 transformed cells is associated with checkpoint induction

**Fig 7:**
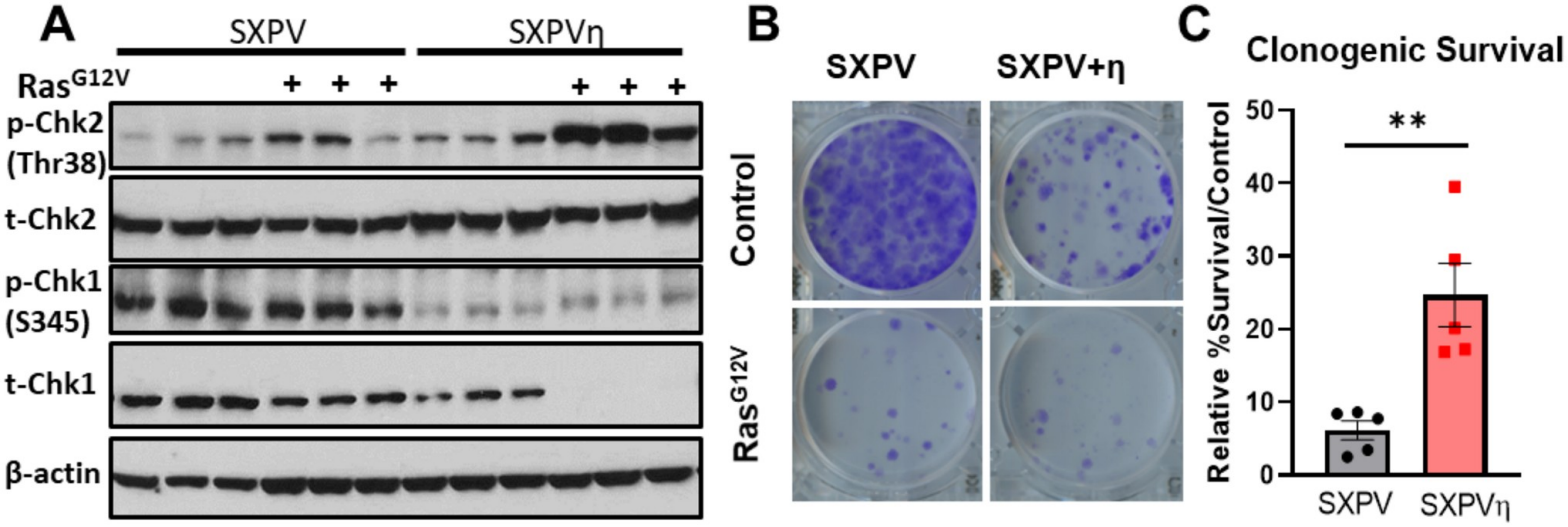
Oncogenic Ras^G12V^-induced DNA polymerase depletion is associated with altered checkpoint signaling. (A). Immunoblot analysis of control or Ras^G12V^-infected SXPV/SXPVη cells for indicated checkpoint proteins. All three biological replicates are shown. (B). Representative crystal violet staining of control-infected or Ras^G12V^-infected SXPV/SXPVη cells at Day 10. Each cell line was selected with puromycin for 2 days after retroviral infection, followed by seeding at 1000 cells per well (in triplicate). Media was replaced every 2–3 days. After 10 days, colonies were fixed with methanol and stained with crystal violet. (C). Quantitation of survival in Ras^G12V^-infected SXPV/SXPVη cells at Day 10, relative to respective controls. The intensity of crystal violet staining in each well was quantified using 590 nm absorbance after destaining. Raw values/well were normalized to a control well with no cells. Survival percentages were calculated as the ratio of normalized mean values from Ras^G12V^-infected wells to mean values from control-infected wells. Data represent mean +/- SEM of five biological replicates.

Oncogene-induced replication stress is well known to increase markers of DNA damage; therefore, we examined activation of the DNA damage response in both cell lines. In SXPVη cells (with polymerase degradation), we observed a very robust increase in Thr68 phospho-Chk2 after 8 days of Ras^G12V^ OE, accompanied by a dramatic depletion of total Chk1 protein expression (Fig 7A). This observation is consistent with previous findings that cells suppress and deplete total Chk1 protein during genotoxic stress and nutrient deprivation via the senescence or the autophagy program [57–60], perhaps as a mechanism of terminating the S phase checkpoint [61]. In contrast, after 8 days of Ras^G12V^ OE, SXPV cells (without polymerase degradation) showed less robust activation of Thr68 phospho-Chk2, while Chk1 was constitutively phosphorylated (Ser 345) (Fig 7A). Thus, polymerase depletion in response to Ras^G12V^ OE is correlated not only with altered fork progression, but also with differential checkpoint activation. Finally, we examined the ability of cells to survive prolonged oncogene overexpression. After 10 days of Ras^G12V^ OE, we measured a significantly greater survival of SXPVη cells, relative to control infected cells, as compared to SXPV cells (Fig 7B and 7C). Thus, the presence of Pol η appears to improve the ability of cells to survive Ras^G12V^ OE. Perhaps, DNA polymerase depletion in response to Ras^G12V^ signaling allows cells to adapt to oncogene-induced replication stress by limiting replication fork progression and enforcing checkpoint activation, which ultimately improves cell survival.

## Discussion

DNA replication is a crucial phase of mitotic cell growth, as it represents the time during which the genome is most vulnerable to damage and mutation. DNA polymerases are integral to all replication process, including ongoing fork elongation, fork restart, and fork repair. Despite their central role in replication, much remains unclear about the regulation of DNA polymerases in response to oncogene-activation. In this study, we investigated the consequences of mutant H-Ras^G12V^ overexpression on DNA polymerase expression levels in human cells, focusing on a subset of polymerases known to be engaged at the replication fork. To our knowledge, our study is the first to report the robust cellular response to oncogenic Ras activation that depletes multiple DNA polymerases. Our results suggest that distinct mechanisms regulate replicative *versus* specialized DNA polymerases in response to Ras^G12V^ overexpression: replicative polymerases are primarily regulated at the gene level (Figs 2 and 4), while specialized polymerases are primarily regulated at the protein level (Figs 2 and 3). Taken together, our study has uncovered a novel cellular response to oncogene activation that is distinct from the response to replication stress induced by DNA damaging agents or drugs, from the perspective of DNA polymerase regulation. This discovery has important implications for how human cells regulate DNA polymerases to limit DNA synthesis in response to endogenous replication stress.

Oncogenic Ras^G12V^ overexpression in nontumorigenic cells induces an initial hyperproliferative response, followed by cellular senescence [1, 2, 62]. This response is accompanied by markers of replication stress, such as decreased fork elongation rate and increased DNA damage. Many mechanisms have been identified that contribute to oncogene-induced replication stress, including dysregulated replication initiation, altered nucleotide metabolism, transcriptional R-loops, and oxidative stress [1, 2, 5, 15, 63]. Here, we demonstrate a dramatic downregulation/degradation of DNA polymerases that are required for ongoing fork elongation, which we propose might contribute directly to oncogene-induced replication stress. Using SV40 transformed cell lines, we observed that cells undergoing oncogene-induced polymerase degradation display the decreased replication fork progression characteristic of replication stress, whereas fork lengths do not change in XPV30R0 cells without DNA polymerase degradation (Fig 6). After 8 days of Ras^G12V^ OE, we observed that cell populations with reduced replicative polymerase levels retain EdU^+^, S-phase cells, albeit with slowed replication forks (Fig 6). Our observation that levels of the Pol α catalytic subunit were low to undetectable in SV40 Ras^G12V^ OE cells, despite retaining EdU^+^ cells, was unexpected and warrants further investigation. One possible explanation of our results is that Pol α formation of initiator DNA from RNA primers created by the Pol α primase subunits is not an absolute requirement for ongoing lagging strand synthesis, and that the Pol δ holoenzyme can extend RNA primers directly. This hypothesis is based on a recent *in vitro* study of eukaryotic replisomes [64]. Using single molecule analyses and purified *S*. *cerevisiae* replication proteins, Lewis *et al*. propose that Pol α DNA synthesis activity is not absolutely required during processive leading/lagging strand replications; rather, only the primase activity of Pol α-primase is required [64]. We did not examine levels of the primase subunits of the Pol α holoenzyme before and after Ras^G12V^ OE. An alternative (not mutually exclusive) hypothesis to explain our results is that PrimPol is activated in response to Ras^G12V^ OE to compensate for the lost functions of DNA Pol α during ongoing fork elongation. We did not examine PrimPol levels in our study; however, under stress condition, the PrimPol enzyme is known to assist in DNA replication [65–68]. Lastly, we measured robust Chk2 activation concomitant with Chk1 depletion in cells undergoing oncogene-induced polymerase degradation (Fig 6). Pol α and Pol κ both mediate *ATR* activation via the 9-1-1 complex [69, 70], and we show that both polymerases are targets for Ras-induced depletion. Perhaps, the depletion of polymerases associated with the replication fork and required for *ATR* activation contributes to cell cycle checkpoint enforcement.

An *H-RAS*-induced senescence-associated protein degradation (SAPD) response has been reported, in which proteins are selectively degraded in an *ERK* and proteasome dependent process [71]. Consistent with this report, we demonstrate that proteasome inhibition partially rescued polymerase proteins, suggesting that SAPD may be responsible, in part, for Ras^G12V^-induced polymerase depletion. Pol η is heavily targeted for depletion and re-localization after DNA damage through several post-translational modifications, including phosphorylation, ubiquitination and SUMOylation [46, 72–75]. Pol η also can be regulated indirectly via Rad18 phosphorylation through *JNK* signaling, another major pathway downstream of RAS [72]. The *RAS* family of proto-oncogenes (K-, H, N-Ras) mediate vital cellular processes such as growth, survival, metabolism, through several mitogenic pathways [17]. More experiments are required to unravel the complex signaling pathways underlying DNA polymerase regulation downstream of the *RAS* signaling axes and advance our understanding of the cellular replication stress response to oncogene activation.

Previous studies have provided indirect evidence that Pol η or κ depletion may result in senescence phenotypes [51, 52, 76]. Here, we report that *POLH* knockdown in *hTERT* BJ5a cells directly induces a senescent-like phenotype (Fig 5). Pol η depletion also significantly reduced Pol α expression, but unlike Ras OE, levels of other polymerases such as Pols κ and δ were not altered. These results suggest that DNA polymerase depletion may be dependent on the mechanisms inducing senescence. One conceivable mechanism that should be explored further is that the cellular depletion of Pol η induces senescence by increasing replication stress [31].

The coordinated degradation of replicative polymerases has been observed in fission yeast as a response to replication stress induced genetically in ΔSwi1 cells (ortholog of timeless) [77]. In that study, the forced accumulation of replication proteins was accompanied by excessive mitotic aberrations, suggesting that the degradation of replisome components plays a critical role in maintaining genome stability. We observed that Ras^G12V^ overexpression significantly downregulated the Pol α catalytic subunit and the Pol δ catalytic subunit. Extensive genetic studies using an *S*. *cerevisiae* model demonstrated that reduced expression of either Pol α or Pol δ induces substantially elevated rates of chromosomal loss and instability [78]. Extrapolating from this yeast model to our study here, one could surmise that a temporal window of increased susceptibility to genome instability exists after oncogene activation. The Pol δ accessory subunit, p68 (*POLD3*) has a major effect on Pol δ’s PCNA binding affinity [79] and mediates Pol δ’s retention in the replisome [64]. Our discovery that p68 also is a target for Ras-induced degradation is provocative, given that p68 (*POLD*3) is required for break-induced replication [34, 80] and for mitosis-associated DNA synthesis [81].

Our discovery of Ras^G12V^-induced DNA polymerase depletion has significant implications for understanding genome instability during carcinogenesis. Overexpression of oncogenes such as Ras^G12V^ and Cyclin E induce a unique landscape of CFS breakage [19]. Fragile sites may contain difficult to replicate sequences (DiToRS), such as microsatellite sequences, non-B DNA structures, and R-loops, that are prone to double strand breaks (reviewed in [28, 63]. Specialized polymerases Pols η and κ are able to efficiently replicate DiToRS [30, 82], and Pol η-deficient cells display elevated levels of replication stress and CFS breakage [27, 31, 83]. Our findings here of Ras-induced Pol η and κ depletion raise the possibility that altered polymerase levels in cells undergoing oncogene activation contribute to genome instability such as the expression of CFS during neoplastic progression. Moreover, it remains unclear how depletion or altered regulation of DNA polymerases during oncogene activation impacts DNA replication and repair mechanisms. One intriguing question is whether different oncogenes have differential impacts on polymerase expression levels, and whether this regulation is based on oncogene-induced replication stress intermediates, such as depleted nucleotides or unusual DNA secondary structures. For example, hydroxyurea induces replication stress by depleting nucleotide pools and requires Pol κ for replication stress tolerance [76]. Recent studies have shown that Pol κ is preferentially utilized in Cyclin E/CDK2-mediated oncogenic stress [35], and the level of nuclear Pol κ is increased after treatment with *BRAF*, *MEK*, or *ERK* inhibitors [84]. In contrast, another replication stress inducer, aphidicolin, stalls DNA replication by inhibiting replicative polymerases and requires Pol η for tolerance (27,31), and Pol η is utilized in Myc-mediated oncogenic stress (36). Together, current evidence supports the supposition that specific DNA polymerases are better suited for alleviating different causes of replication stress. Thus, elucidating the exact replication intermediates and signaling pathways that mediate DNA polymerase regulation, recruitment, and fork progression will be crucial to understanding the mechanisms underlying oncogene-induced replication stress and the roles of DNA polymerases in promoting tumorigenesis.

## Supporting information

S1 Table List of key resources. (PDF)

S1 Fig Replicates for Ras-infected BJ5a cells treated with MG132 inhibitor. Immunoblot analyses of polymerase levels at indicated days after transduction/selection with control or Ras^G12V^ vectors. BJ5a cells were either treated with DMSO or MG132 (10μM) for 4 hours prior to harvesting. Red values are quantification of polymerase levels, normalized to Control or Ras^G12V^ treated with DMSO.
(TIF)

S1 File Original, uncropped images for all immunoblots. (PDF)
