# Supplementary material for: Overexpression of oncogenic H-Ras in hTERT-immortalized and SV40-transformed human cells targets replicative and specialized DNA polymerases for depletion
Source: PLoS One. 2021 May 7;16(5):e0251188. doi: 10.1371/journal.pone.0251188 (PMC8104423; doi:10.1371/journal.pone.0251188)
Supplement: S1 Table — (PDF) [file pone.0251188.s001.pdf]

Reagent Type	Designation	Source or Reference	Identifiers	Additional Information
Cell line	hTERT-BJ-5a	ATCC	CRL-4001	
Cell line	SV40 XPV/XPVη	Dr. Jean-Sebastian Hoffman		
Cell line	IMR90	K. Aird laboratory ATCC	CCL-186	
Cell line	Phoenix cells (QNX)	K. Aird laboratory		
Cell line	HEK293FT	K.Aird laboratory Thermofisher	R70007	
Antibody	Rabbit anti-Polη	Cell signaling	13848S	WB 1:500-1:1000
Antibody	Mouse anti-Polκ	Santa Cruz	sc-16667	WB 1:1000
Antibody	Rabbit anti-Polβ	Abcam	ab1831	WB 1:4000
Antibody	Mouse anti-POLD3	Abnova	H00010714-m01	WB 1:1000
Antibody	Mouse anti-PODL2	Abcam	ab38338	WB 1:1000
Antibody	Rabbit anti-POLD1	Abcam	ab186407	WB 1:10,000
Antibody	Mouse anti-Polα	Santa Cruz	sc-373884	WB 1:200
Antibody	Mouse anti-β-actin	Santa Cruz	sc-47778	WB 1:10,000
Antibody	Rabbit anti-CDKN2A/p16	Abcam	ab108349	WB 1:1000
Antibody	Rabbit anti-p44/42 MAPK	Cell signaling	9102S	WB 1:1000
Antibody	Rabbit anti-phospho p44/42	Cell signaling	4370S	WB 1:12,000
Antibody	Rabbit anti-laminB1	Abcam	ab16048	WB 1:8000
Antibody	Rabbit anti-phospho Chk1 (S345)	Cell signaling	2348S	WB 1:1000
Antibody	Rabbit anti-total Chk1	Santa Cruz	sc-8408	WB 1:100
Antibody	Rabbit anti-phospho Chk2 (Thr68)	Cell signaling	2197S	WB 1:1000
Antibody	Rabbit anti-total Chk2	Abcam	ab32148	WB 1:8000
Antibody	Mouse anti-Ras	Milipore	05-516	WB 1:20,000
Antibody	Mouse anti-RRM2	Santa Cruz	sc-398294	WB 1:1000
Antibody	Rabbit-anti-53BP1	Bethyl	A300-272A	IF 1:200
Antibody	Goat-anti-rabbit	Santa Cruz	sc-2004	WB 1:20,000
Antibody	Goat-anti-mouse	Santa Cruz	sc-2005	WB 1:20,000

Antibody	Goat-anti-mouse Alexa 488	Thermofisher	A32723	Fiber 1:1000
Antibody	Goat-anti-rat Alexa 594	Thermofisher	A11007	Fiber 1:1000
Constructs	pLKO.1 Control lentiviral vector	Addgene	8453	
Constructs	pLKO.1 shp16	Sigma-Aldrich	TRCN0000010482	
Constructs	pLKO.1 shPOLH #2	Sigma-Aldrich	TRCN0000053008	
Constructs	pLKO.1 shPOLH #1	Sigma-Aldrich	TRCN0000053010	
Constructs	pBABE control retroviral vector	K. Aird lab/Addgene	1764	35μg/10cm plate
Constructs	pBABE HRASG12V	K. Aird lab/Addgene	12274	35ug/10cm plate
Taqman primers	POLH	Thermofisher	Hs00982625	FAM
Taqman primers	POLK	Thermofisher	Hs00211963	FAM
Taqman primers	POLA1	Thermofisher	Hs00213524	FAM
Taqman primers	POLE1	Thermofisher	Hs00923954	VIC
Taqman primers	POLD1	Thermofisher	Hs01100821	FAM
Taqman primers	POLD3	Thermofisher	Hs01075255	VIC
Taqman primers	POLB	Thermofisher	Hs01099715	VIC
Taqman primers	18S	Thermofisher	Hs03928985	VIC
Taqman primers	CDKN2A	Thermofisher	Hs00923894	FAM
Taqman primers	LAMINB1	Thermofisher	Hs01059210	VIC
Chemical reagent/Drug	Polybrene	Sigma-Aldrich	H9268	
Chemical reagent/Drug	MG132	Sigma-Aldrich	M7449	
Chemical reagent/Drug	X-gal	Sigma-Aldrich	B4252	
Chemical reagent/Drug	Puromycin	Gibco/Life-technologies	A11138-02	
Chemical reagent/Drug	IdU	Abcam	ab-6326	
Chemical reagent/Drug	CldU	BD	347580	
Chemical reagent/Drug	EdU	Lumiprobe	10540	
Chemical reagent/Drug	Sulfo-cy5 azide	Lumiprobe	B3030	
Chemical reagent/Drug	Sulfo-FAM azide	Lumiprobe	B4130	
Chemical reagent/Drug	Propidium iodide	Sigma-Aldrich	P4170	
Chemical reagent/Drug	Mounting medium with DAPI	Vectorlabs	H-1200	

Chemical reagent/Drug	Crystal violet	Fisher	C581-25	
Chemical reagent/Drug	Ponceau S	Sigma-Aldrich	P7170	
Chemical reagent/Drug	Paraformaldehyde	Sigma-Aldrich	158127	
Chemical reagent/Drug	Formaldehyde	Sigma-Aldrich	F8775	
Chemical reagent/Drug	Glutaraldehyde	Sigma-Aldrich	G5882	
Chemical reagent/Drug	Halt prot. And pho. Inhibitors	Thermofisher	78442	
Chemical reagent/Drug	PMSF	Santa Cruz	CAS 329-98-6	
Chemical reagent/Drug	BSA	Sigma-Aldrich	A9647	
Chemical reagent/Drug	non-fat milk	Bio-rad	170-6404	
Chemical reagent/Drug	Tween	Fisher	BP337	
Chemical reagent/Drug	Triton-X	Fisher	BP151	
Chemical reagent/Drug	copper sulate pentahydrate	Sigma-Aldrich	209198	
Chemical reagent/Drug	L-ascorbic acid	Sigma-Aldrich	A4403	
Chemical reagent/Drug	RNAse	Sigma-Aldrich	R5000	
Chemical reagent/Drug	Acetic acid	Fisher	A38	
Chemical reagent/Drug	Methanol	Fisher	A412	
Transfection reagent	lipofectamine 2000	Life-technologies	11668019	1:1 DNA
Transfection reagent	Polyethylenimine	Alfa Aesar	43896.01	1:1 DNA
Commercial assay or kit	qScript cDNA synthesis kit	Quanta Biosciences	95047-100	
Commercial assay or kit	PerfeCTa Fast Mix II, Low Rox	Quanta Biosciences	95120-012	
Commercial assay or kit	DC Bio-rad protein assay	Bio-rad	5000112	
Commercial assay or kit	4X LDS sample buffer	Life-technologies	NP0007	
Commercial assay or kit	10X Reducing agent	Life-technologies	NP0009	
Commercial assay or kit	RNeasy plus mini kit	Qiagen	74134	
Commercial assay or kit	NuPAGE pre-casted gels	Life-technologies	NP series	

Commercial assay or kit	Amersham ECL Prime	GE Healthcare	RPN2232	
Commercial assay or kit	Pierce ECL western blot substrate	Thermofisher	80196, 32106	
Miscellaneous reagents	low-melting agarose	Bio-rad	1613111	
Miscellaneous reagents	0.2µm PVDF membranes	GE Healthcare	10600021	
Miscellaneous reagents	CL-Xposure Films	Thermofisher	34090	
Culture media	DMEM High Glucose	Corning/Fisher Sci	10-013-CV	
Culture media	DMEM	Sigma-Aldrich	D7777	
Culture media	DMEM/F12	Sigma-Aldrich	D9785	
Culture media	minimal essential amino acids	Sigma-Aldrich	M0643	
Culture media	Medium 199	Corning/Fisher Sci	10-060-CVR	
Culture media	Sodium bicarbonate	Sigma-Aldrich	144-55-8	
Culture media	HEPES	Sigma-Aldrich	H3375	
Culture media	Gentimicin	Life-technologies	30-005-CR	
Culture media	FBS	Hyclone/GE healthcare	SH30071.03	
Culture media	PBS	Millipore/Sigma	P3813	
