# Supplementary figures and images for: Overexpression of oncogenic H-Ras in hTERT-immortalized and SV40-transformed human cells targets replicative and specialized DNA polymerases for depletion
Source: PLoS One. 2021 May 7;16(5):e0251188. doi: 10.1371/journal.pone.0251188 (PMC8104423; doi:10.1371/journal.pone.0251188)

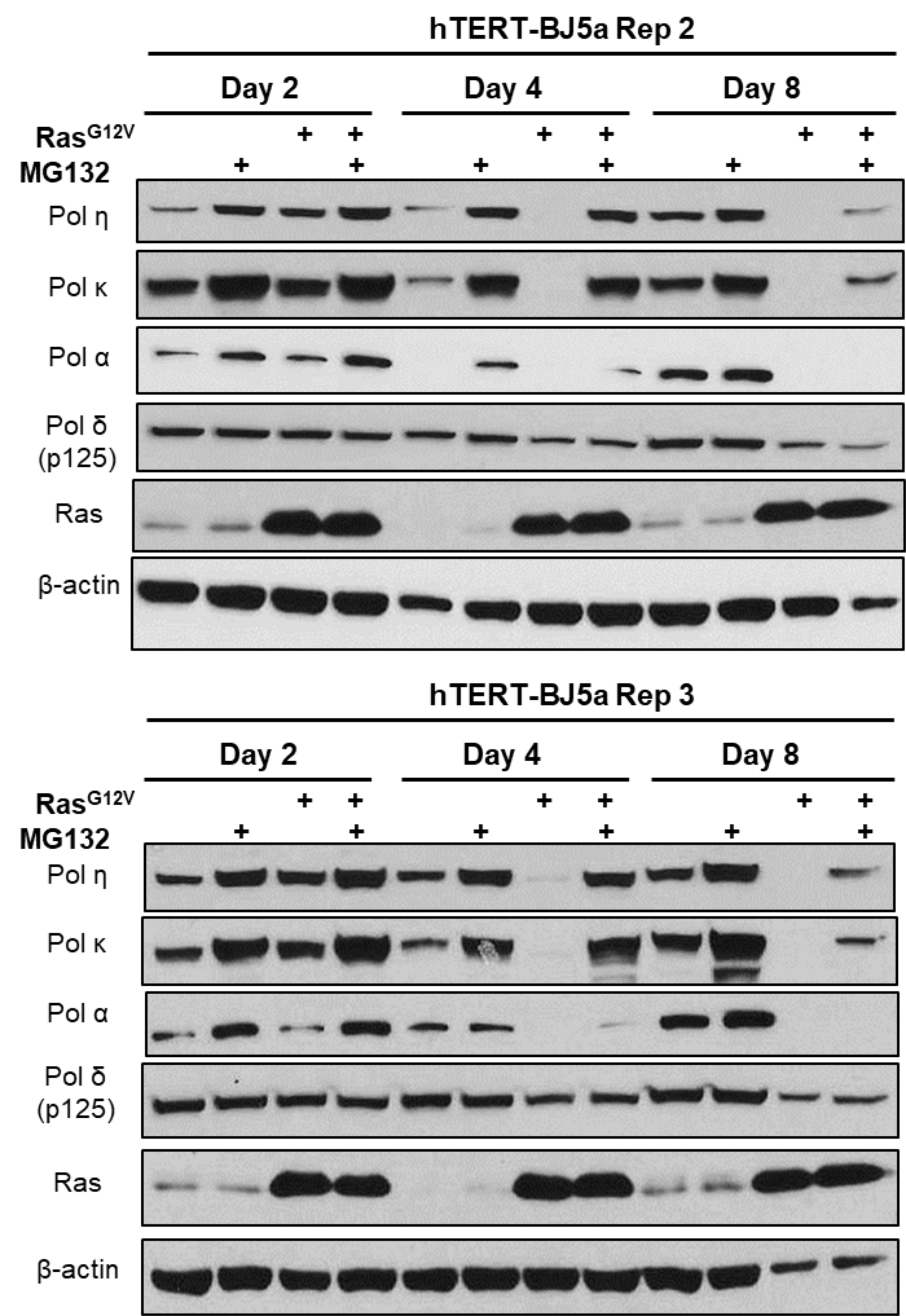

Supplement: S1 Fig — Immunoblot analyses of polymerase levels at indicated days after transduction/selection with control or RasG12V vectors. BJ5a cells were either treated with DMSO or MG132 (10μM) for 4 hours prior to harvesting. Red values are quantification of polymerase levels, normalized to Control or RasG12V treated with DMSO. (TIF) [file pone.0251188.s002.tif]
