# Supplementary material for: Overexpression of oncogenic H-Ras in hTERT-immortalized and SV40-transformed human cells targets replicative and specialized DNA polymerases for depletion
Source: PLoS One. 2021 May 7;16(5):e0251188. doi: 10.1371/journal.pone.0251188 (PMC8104423; doi:10.1371/journal.pone.0251188)
Supplement: S1 File — (PDF) [file pone.0251188.s003.pdf]

Fig1D

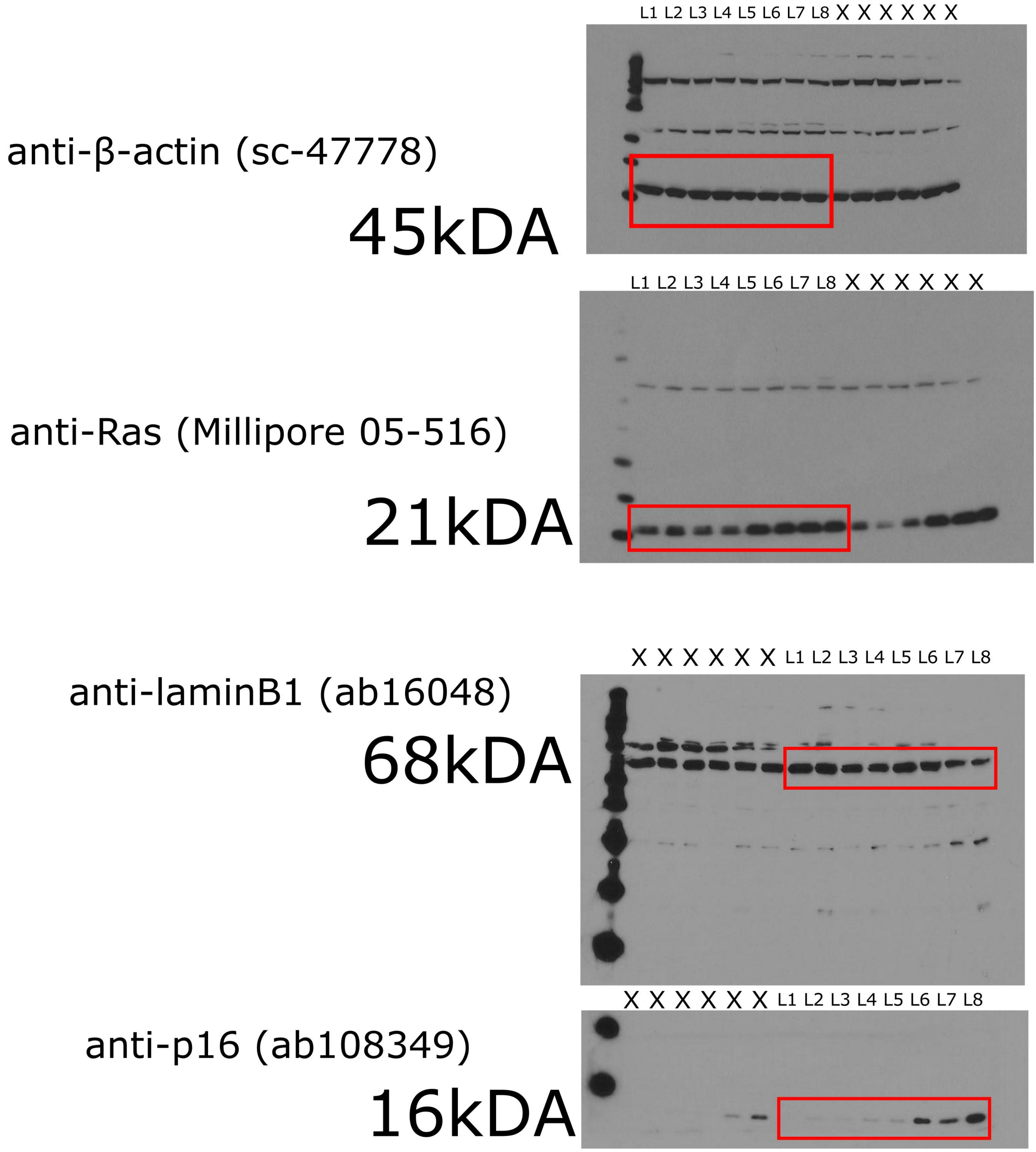

Lane 1: BJ5a cells infected with pbabe control for 2 days
Lane 2: BJ5a cells infected with pbabe control for 4 days
Lane 3: BJ5a cells infected with pbabe control for 6 days
Lane 4: BJ5a cells infected with pbabe control for 8 days
Lane 5: BJ5a cells infected with pbabe HRasG12V for 2 days
Lane 6: BJ5a cells infected with pbabe HRasG12V for 4 days
Lane 7: BJ5a cells infected with pbabe HRasG12V for 6 days
Lane 8: BJ5a cells infected with pbabe HRasG12V for 8 days

Fig2B

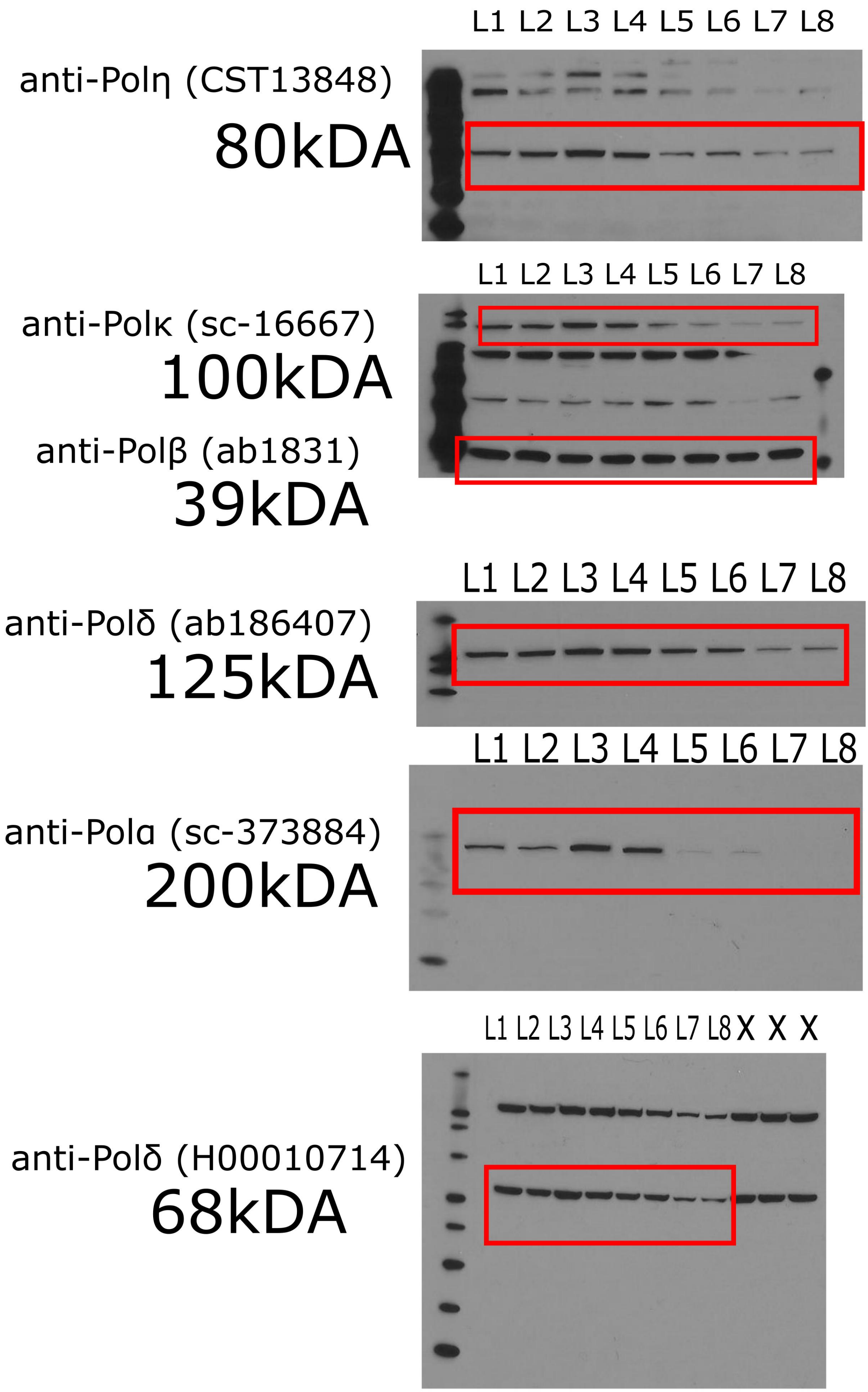

Lane 1: BJ5a cells infected with pbabe control for 2 days
Lane 2: BJ5a cells infected with pbabe control for 4 days
Lane 3: BJ5a cells infected with pbabe control for 6 days
Lane 4: BJ5a cells infected with pbabe control for 8 days
Lane 5: BJ5a cells infected with pbabe HRasG12V for 2 days
Lane 6: BJ5a cells infected with pbabe HRasG12V for 4 days
Lane 7: BJ5a cells infected with pbabe HRasG12V for 6 days
Lane 8: BJ5a cells infected with pbabe HRasG12V for 8 days

Fig2B continued

anti-β-actin (sc-47778)

45kDA

anti-Ras (Millipore 05-516)

21kDA

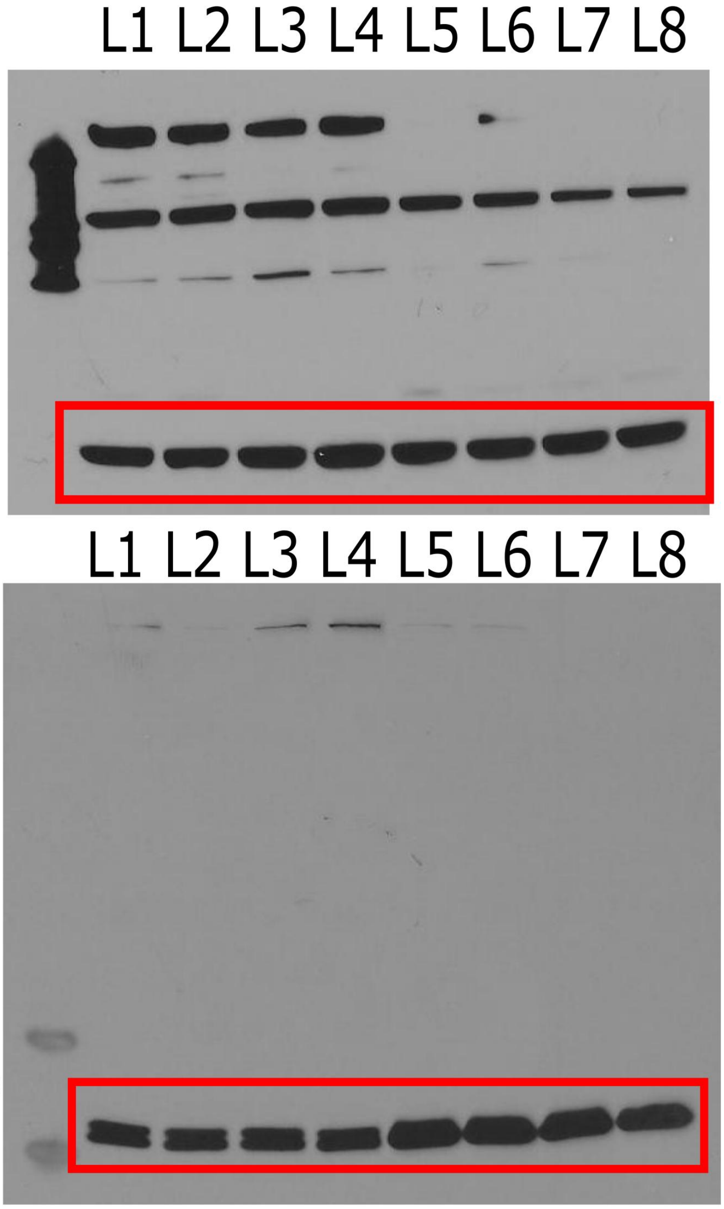

Lane 1: BJ5a cells infected with pbabe control for 2 days
Lane 2: BJ5a cells infected with pbabe control for 4 days
Lane 3: BJ5a cells infected with pbabe control for 6 days
Lane 4: BJ5a cells infected with pbabe control for 8 days
Lane 5: BJ5a cells infected with pbabe HRasG12V for 2 days
Lane 6: BJ5a cells infected with pbabe HRasG12V for 4 days
Lane 7: BJ5a cells infected with pbabe HRasG12V for 6 days
Lane 8: BJ5a cells infected with pbabe HRasG12V for 8 days

Fig1D

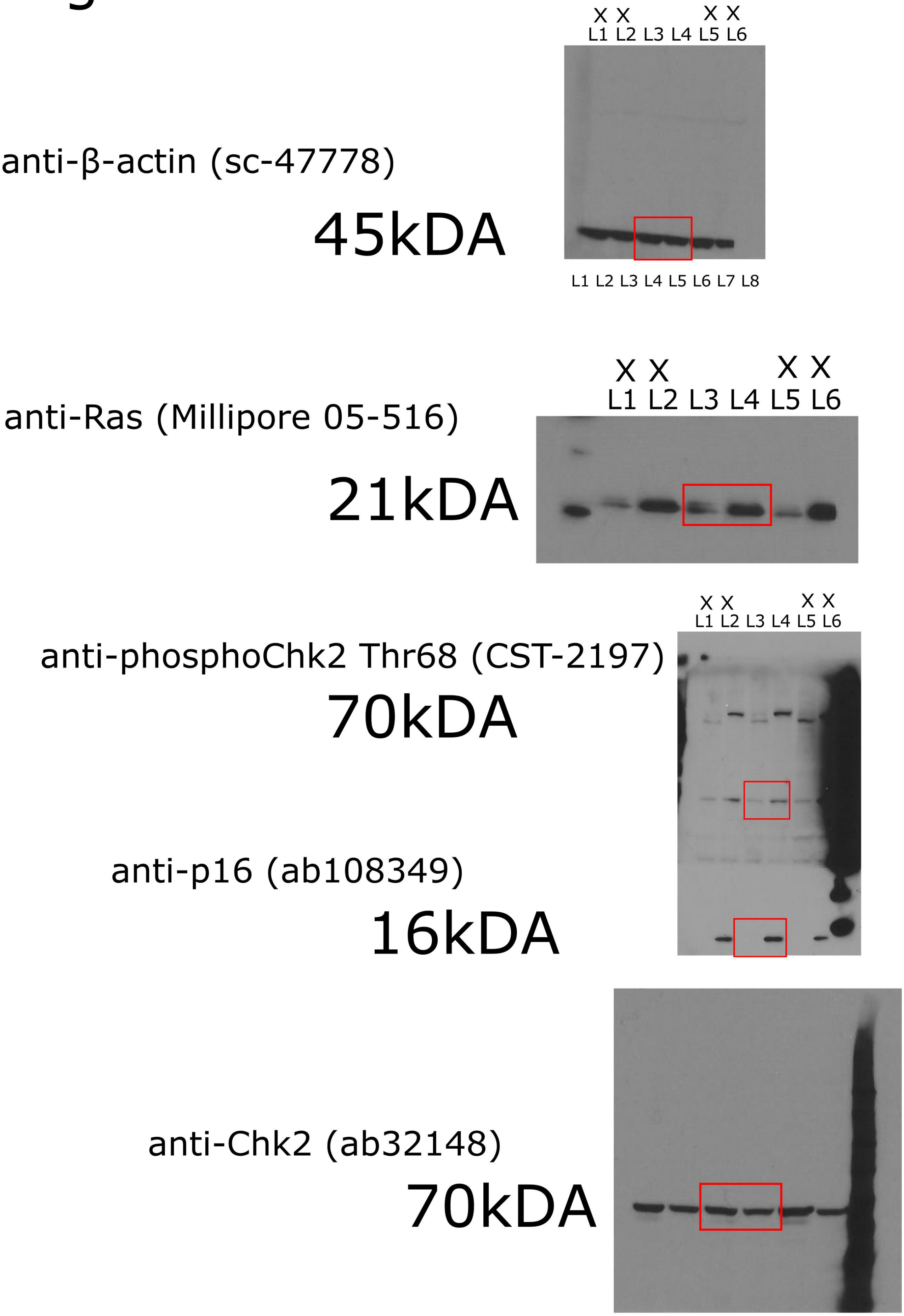

Lane 1: BJ5a cells infected with pbabe control for 8 days rep 1
Lane 2: BJ5a cells infected with pbabe HRasG12V for 8 days rep 1
Lane 3: BJ5a cells infected with pbabe control for 8 days rep 2
Lane 4: BJ5a cells infected with pbabe HRasG12V for 8 days rep 2
Lane 5: BJ5a cells infected with pbabe control for 8 days rep 3
Lane 6: BJ5a cells infected with pbabe HRasG12V for 8 days rep 3

Fig3A

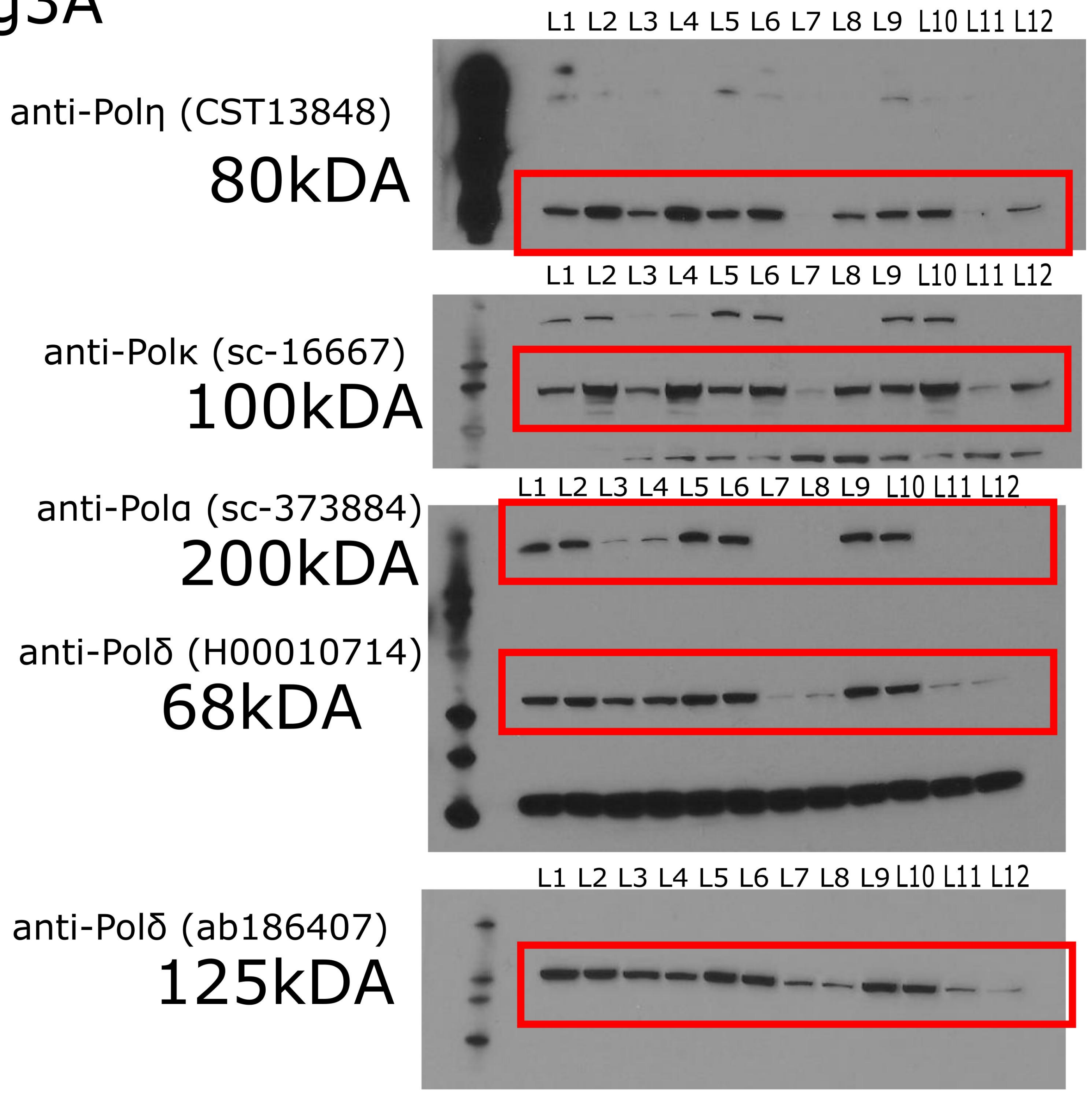

Lane 1: Day 2- BJ5a cells infected with pbabe control treated with DMSO
Lane 2: Day 2- BJ5a cells infected with pbabe control treated with MG132
Lane 3: Day 2- BJ5a cells infected with pbabe HRasG12V treated with DMSO
Lane 4: Day 2- BJ5a cells infected with pbabe HRasG12V treated with MG132
Lane 5: Day 4- BJ5a cells infected with pbabe control treated with DMSO
Lane 6: Day 4- BJ5a cells infected with pbabe control treated with MG132
Lane 7: Day 4- BJ5a cells infected with pbabe HRasG12V treated with DMSO
Lane 8: Day 4- BJ5a cells infected with pbabe HRasG12V treated with MG132
Lane 9: Day 8- BJ5a cells infected with pbabe control treated with DMSO
Lane 10: Day 8- BJ5a cells infected with pbabe control treated with MG132
Lane 11: Day 8- BJ5a cells infected with pbabe HRasG12V treated with DMSO
Lane 12: Day 8- BJ5a cells infected with pbabe HRasG12V treated with MG132

Fig3A continued

anti-Polβ (ab1831)
39kDA

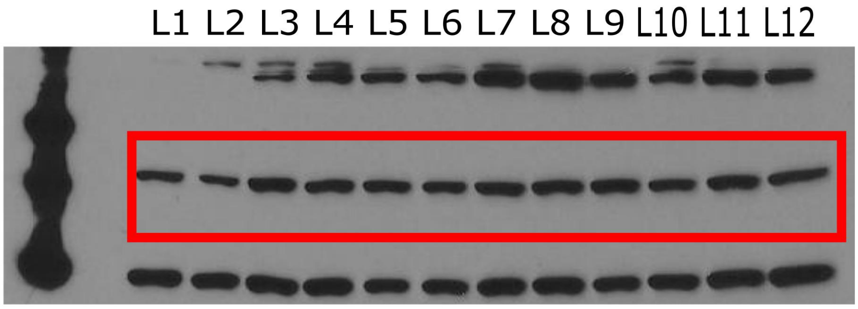

anti-β-actin (sc-47778)
45kDA

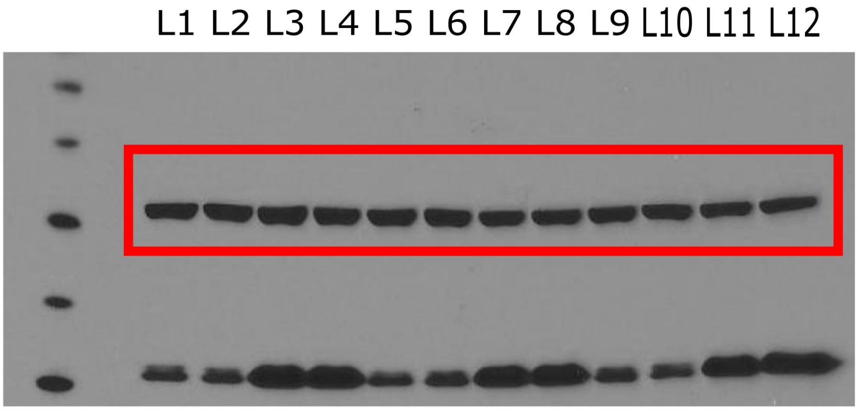

anti-Ras (Millipore 05-516)
21kDA

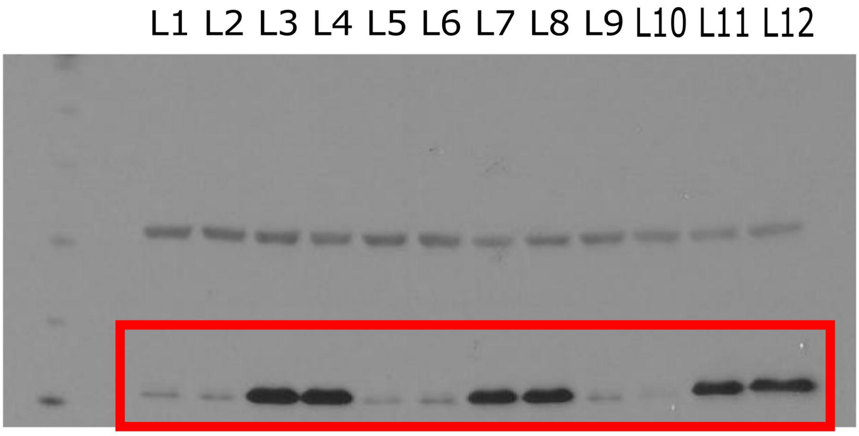

Lane 1: Day 2- BJ5a cells infected with pbabe control treated with DMSO
Lane 2: Day 2- BJ5a cells infected with pbabe control treated with MG132
Lane 3: Day 2- BJ5a cells infected with pbabe HRasG12V treated with DMSO
Lane 4: Day 2- BJ5a cells infected with pbabe HRasG12V treated with MG132
Lane 5: Day 4- BJ5a cells infected with pbabe control treated with DMSO
Lane 6: Day 4- BJ5a cells infected with pbabe control treated with MG132
Lane 7: Day 4- BJ5a cells infected with pbabe HRasG12V treated with DMSO
Lane 8: Day 4- BJ5a cells infected with pbabe HRasG12V treated with MG132
Lane 9: Day 8- BJ5a cells infected with pbabe control treated with DMSO
Lane 10: Day 8- BJ5a cells infected with pbabe control treated with MG132
Lane 11: Day 8- BJ5a cells infected with pbabe HRasG12V treated with DMSO
Lane 12: Day 8- BJ5a cells infected with pbabe HRasG12V treated with MG132

Fig3C

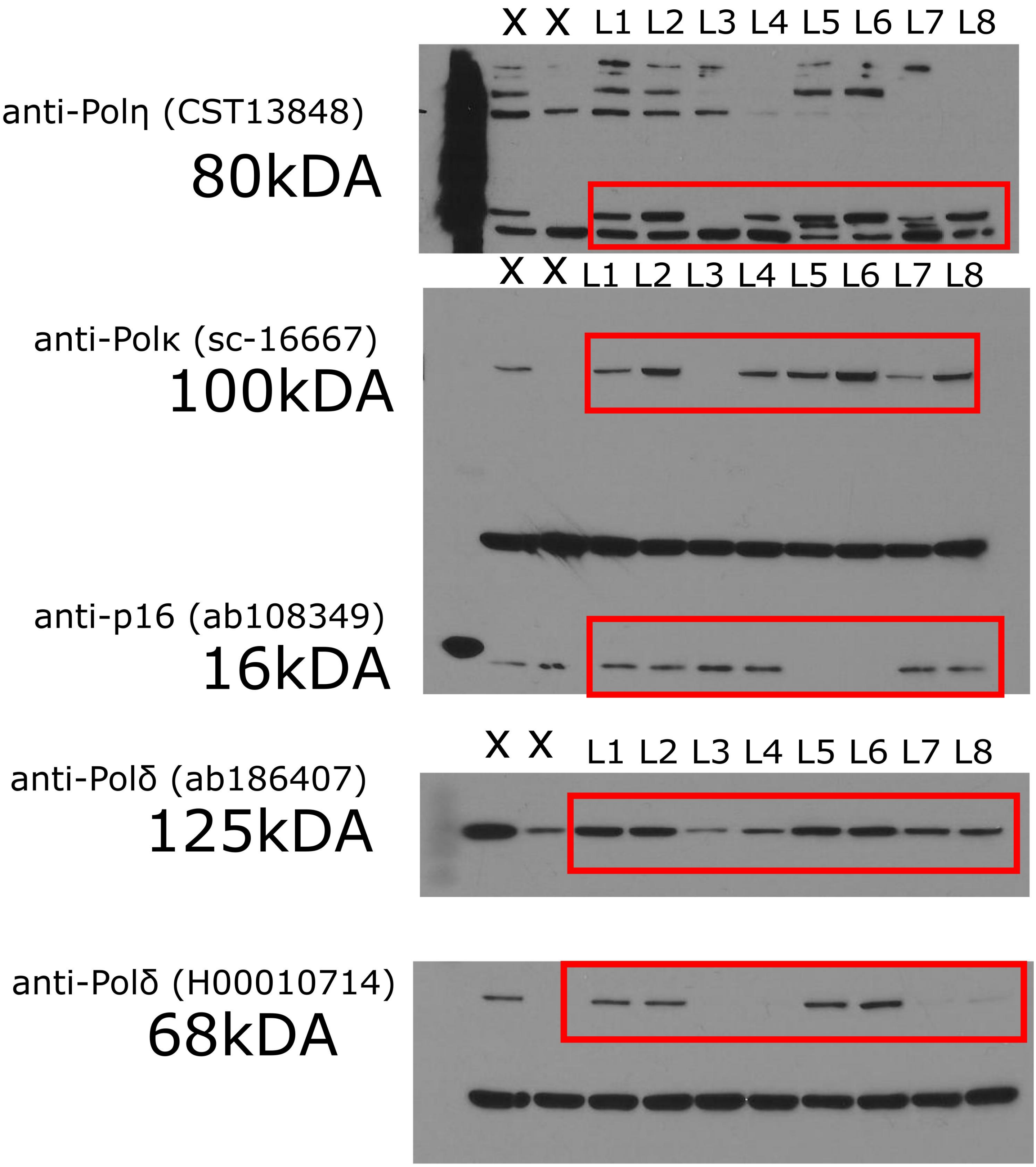

Lane 1: Day 8- IMR90 cells infected with pbabe control treated with DMSO
Lane 2: Day 8- IMR90 cells infected with pbabe control treated with MG132
Lane 3: Day 8- IMR90 cells infected with pbabe HRasG12V treated with DMSO
Lane 4: Day 8- IMR90 cells infected with pbabe HRasG12V treated with MG132
Lane 5: Day 8- BJ5a cells infected with pbabe control treated with DMSO
Lane 6: Day 8- BJ5a cells infected with pbabe control treated with MG132
Lane 7: Day 8- BJ5a cells infected with pbabe HRasG12V treated with DMSO
Lane 8: Day 8- BJ5a cells infected with pbabe HRasG12V treated with MG132

Fig3C continued

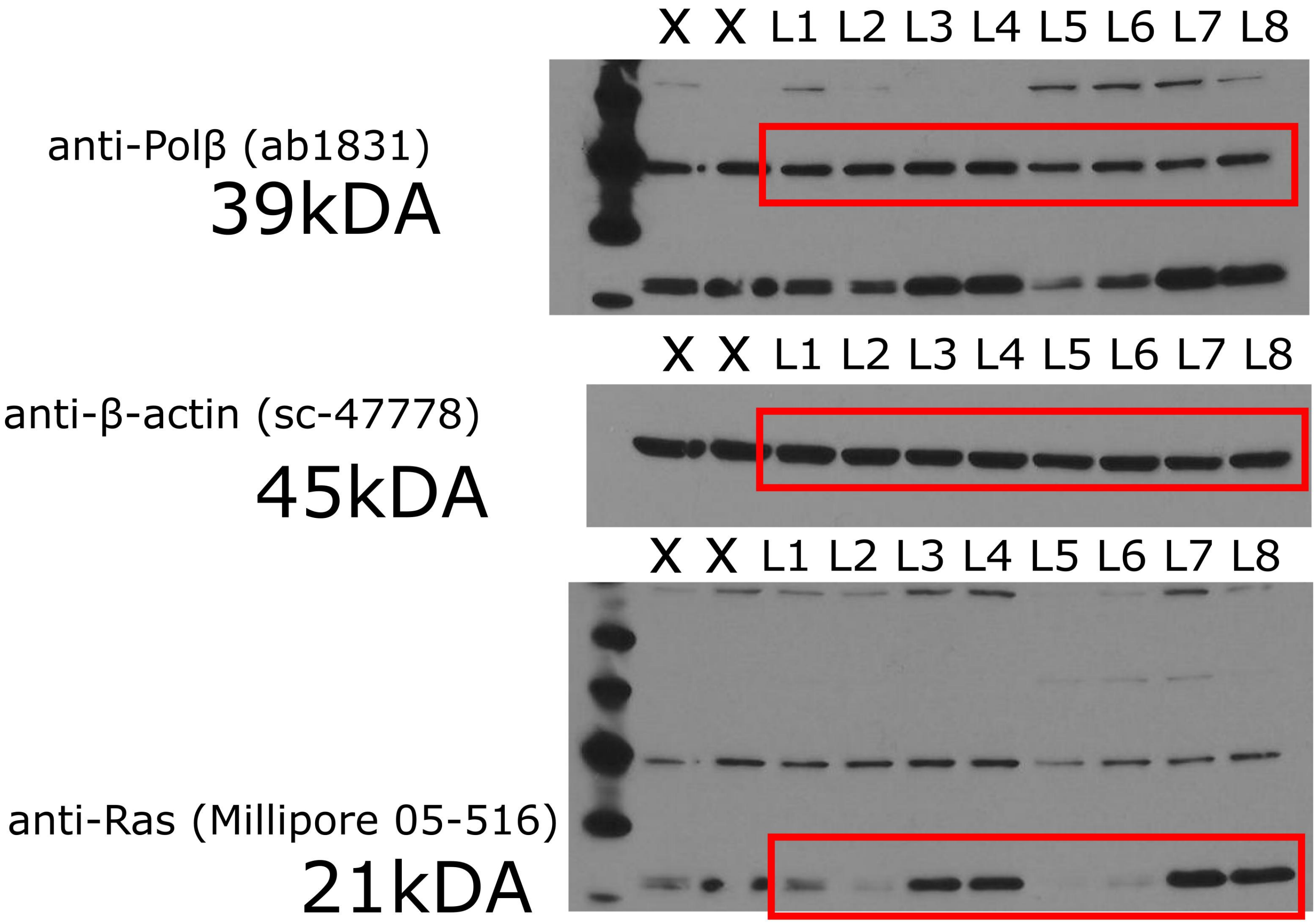

Lane 1: Day 8- IMR90 cells infected with pbabe control treated with DMSO
Lane 2: Day 8- IMR90 cells infected with pbabe control treated with MG132
Lane 3: Day 8- IMR90 cells infected with pbabe HRasG12V treated with DMSO
Lane 4: Day 8- IMR90 cells infected with pbabe HRasG12V treated with MG132
Lane 5: Day 8- BJ5a cells infected with pbabe control treated with DMSO
Lane 6: Day 8- BJ5a cells infected with pbabe control treated with MG132
Lane 7: Day 8- BJ5a cells infected with pbabe HRasG12V treated with DMSO
Lane 8: Day 8- BJ5a cells infected with pbabe HRasG12V treated with MG132

Fig4C

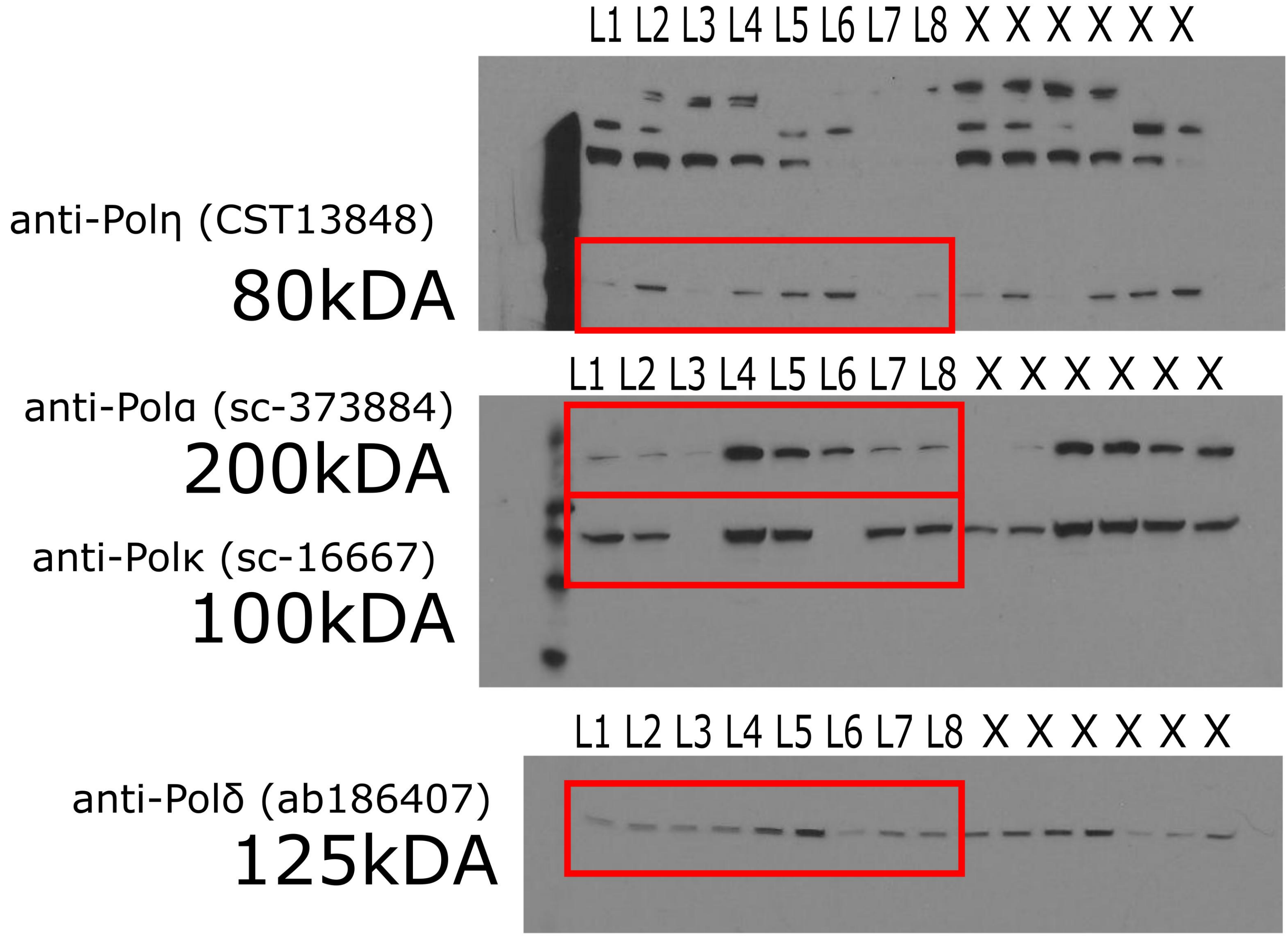

Lane 1: Day 2- BJ5a cells infected with pbabe control and shscrambled
Lane 2: Day 2- BJ5a cells infected with pbabe control and shp16
Lane 3: Day 2- BJ5a cells infected with pbabe HRasG12V and shscrambled
Lane 4: Day 2- BJ5a cells infected with pbabe HRasG12V an shp16
Lane 5: Day 4- BJ5a cells infected with pbabe control and shscrambled
Lane 6: Day 4- BJ5a cells infected with pbabe control tand shp16
Lane 7: Day 4- BJ5a cells infected with pbabe HRasG12V and shscrambled
Lane 8: Day 4- BJ5a cells infected with pbabe HRasG12V shp16

Fig4C continued

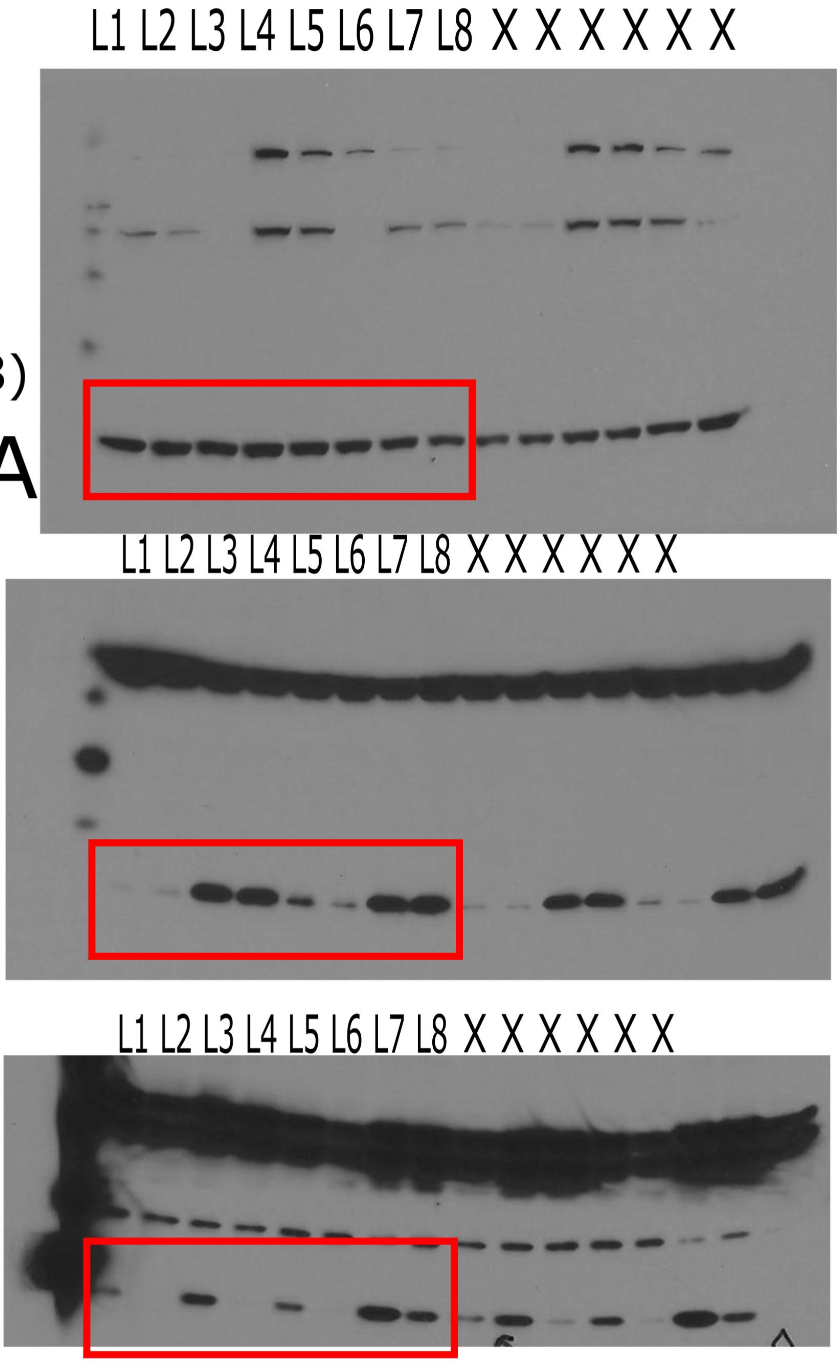

anti-β-actin (sc-47778)
45kDA

anti-Ras (Millipore 05-516)
21kDA

anti-p16 (ab108349)
16kDA

Lane 1: Day 2- BJ5a cells infected with pbabe control and shscrambled
Lane 2: Day 2- BJ5a cells infected with pbabe control and shp16
Lane 3: Day 2- BJ5a cells infected with pbabe HRasG12V and shscrambled
Lane 4: Day 2- BJ5a cells infected with pbabe HRasG12V an shp16
Lane 5: Day 4- BJ5a cells infected with pbabe control and shscrambled
Lane 6: Day 4- BJ5a cells infected with pbabe control tand shp16
Lane 7: Day 4- BJ5a cells infected with pbabe HRasG12V and shscrambled
Lane 8: Day 4- BJ5a cells infected with pbabe HRasG12V shp16

Fig5A

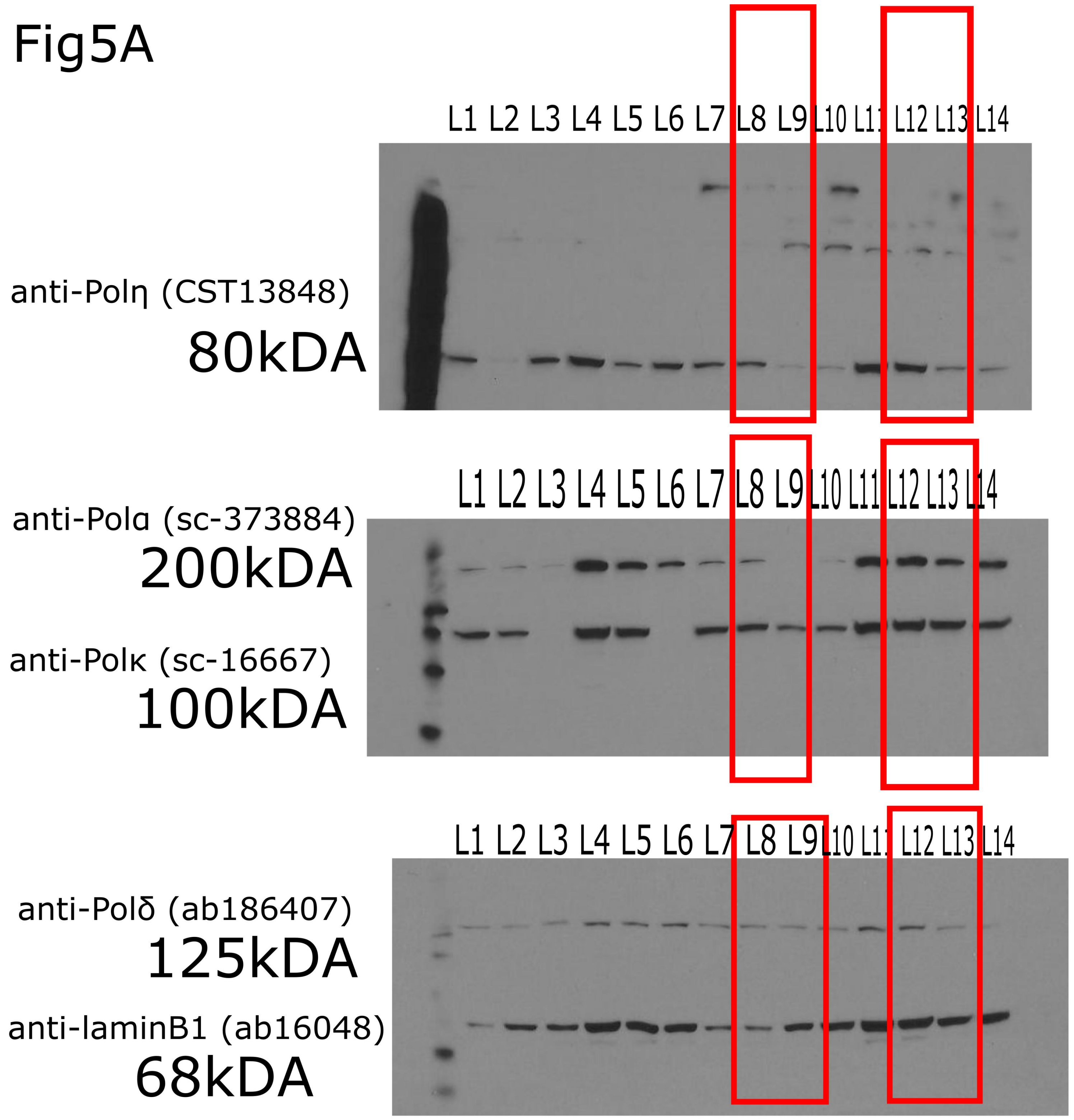

Lane 1: Day 2- BJ5a cells infected with shscrambled- rep 1
Lane 2: Day 2- BJ5a cells infected with shPOLH-1 rep 1
Lane 3: Day 2- BJ5a cells infected with shPOLK
Lane 4: Day 8- BJ5a cells infected with shscrambled- rep 1
Lane 5: Day 8- BJ5a cells infected with POLH-1
Lane 6: Day 8- BJ5a cells infected with shPOLK
Lane 7: Day 2- BJ5a cells infected with shscrambled- rep 2
Lane 8: Day 2- BJ5a cells infected with shscrambled- rep 3
Lane 9: Day 2- BJ5a cells infected with shPOLH-1 rep 2
Lane 10: Day2- BJ5a cells infected with shPOLH-1 rep 3
Lane 11: Day 8- BJ5a cells infected with shscrambled- rep 2
Lane 12: Day 8- BJ5a cells infected with shscrambled- rep 3
Lane 13: Day 8- BJ5a cells infected with shPOLH-1 rep 2
Lane 14: Day8- BJ5a cells infected with shPOLH-1 rep 3

Fig5A continued

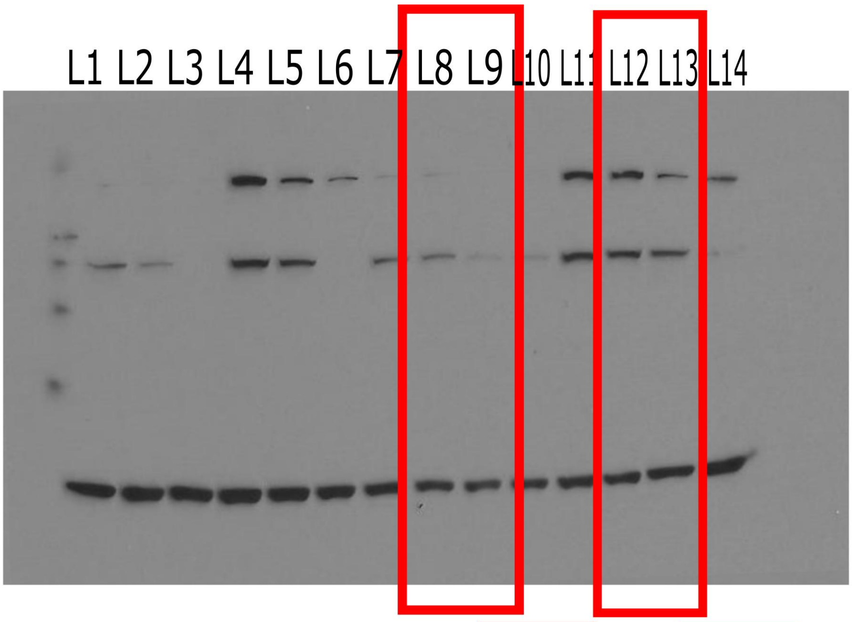

anti-β-actin (sc-47778)
45kDA

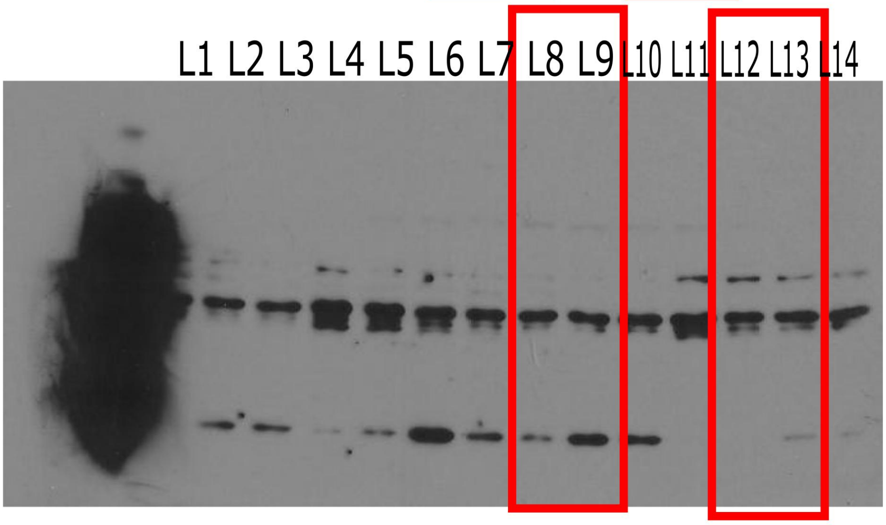

anti-γh2ax phospho S139 (ab11174)
21kDA

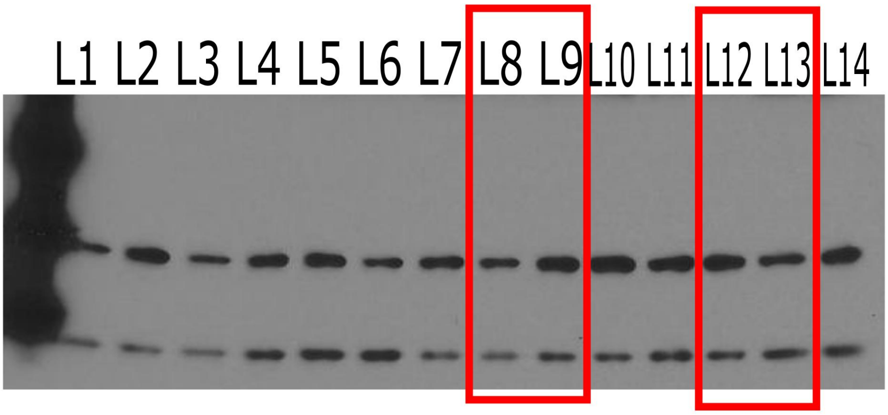

anti-p16 (ab108349)
16kDA

Lane 1: Day 2- BJ5a cells infected with shscrambled- rep 1
Lane 2: Day 2- BJ5a cells infected with shPOLH-1 rep 1
Lane 3: Day 2- BJ5a cells infected with shPOLK
Lane 4: Day 8- BJ5a cells infected with shscrambled- rep 1
Lane 5: Day 8- BJ5a cells infected with POLH-1
Lane 6: Day 8- BJ5a cells infected with shPOLK
Lane 7: Day 2- BJ5a cells infected with shscrambled- rep 2
Lane 8: Day 2- BJ5a cells infected with shscrambled- rep 3
Lane 9: Day 2- BJ5a cells infected with shPOLH-1 rep 2
Lane 10: Day2- BJ5a cells infected with shPOLH-1 rep 3
Lane 11: Day 8- BJ5a cells infected with shscrambled- rep 2
Lane 12: Day 8- BJ5a cells infected with shscrambled- rep 3
Lane 13: Day 8- BJ5a cells infected with shPOLH-1 rep 2
Lane 14: Day8- BJ5a cells infected with shPOLH-1 rep 3

Fig6A

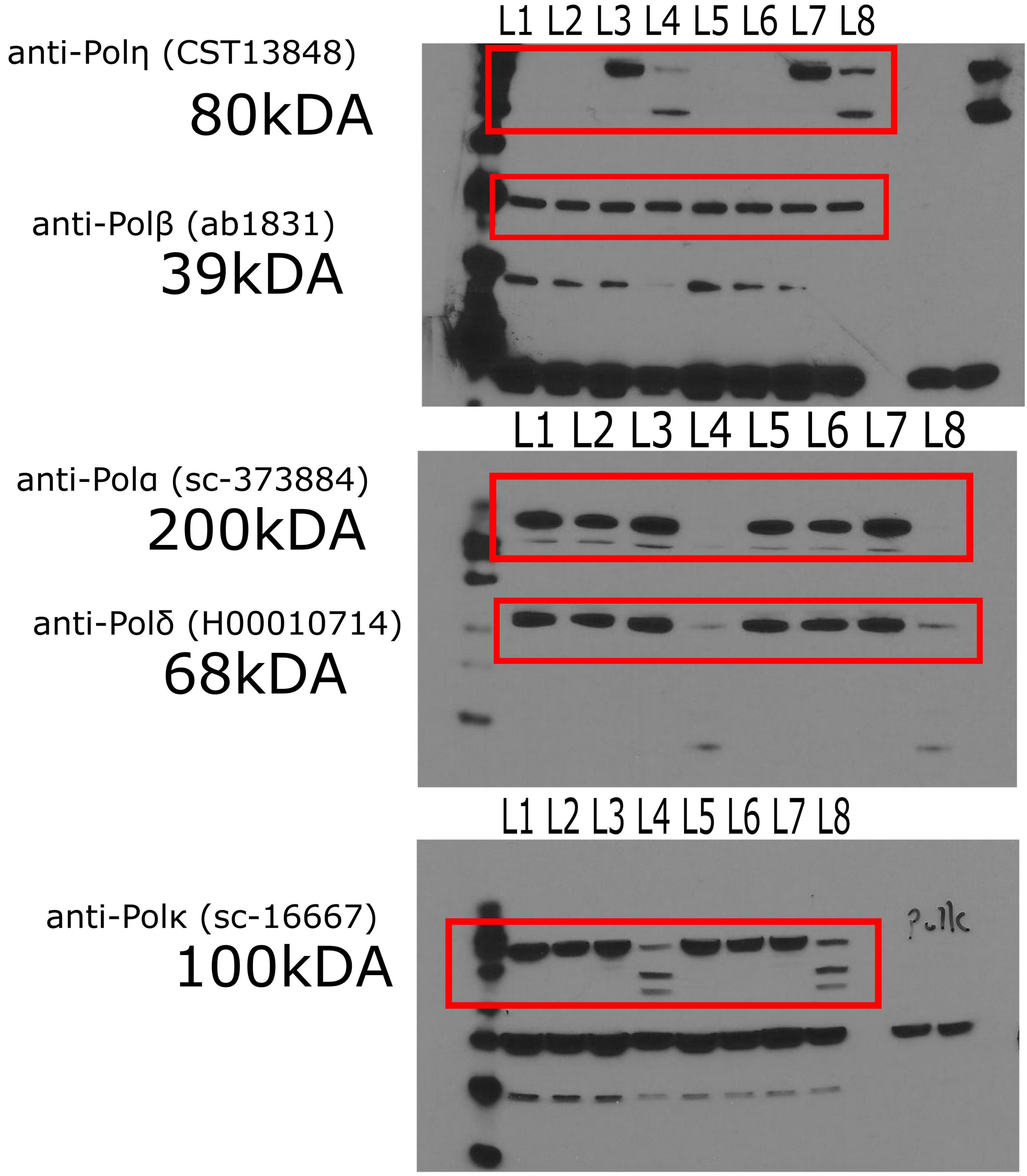

Lane 1: Day 2- SXPV cells infected with pbabe control
Lane 2: Day 2- SXPV cells infected with pbabe HRasG12V
Lane 3: Day 2- SXPV/Polη cells infected with pbabe control
Lane 4: Day 2- SXPV/Polη cells infected with pbabe HRasG12V
Lane 5: Day 8- SXPV cells infected with pbabe control
Lane 6: Day 8- SXPV cells infected with pbabe HRasG12V
Lane 7: Day 8- SXPV/Polη cells infected with pbabe control
Lane 8: Day 8- SXPV/Polη cells infected with pbabe HRasG12V

Fig6A continued

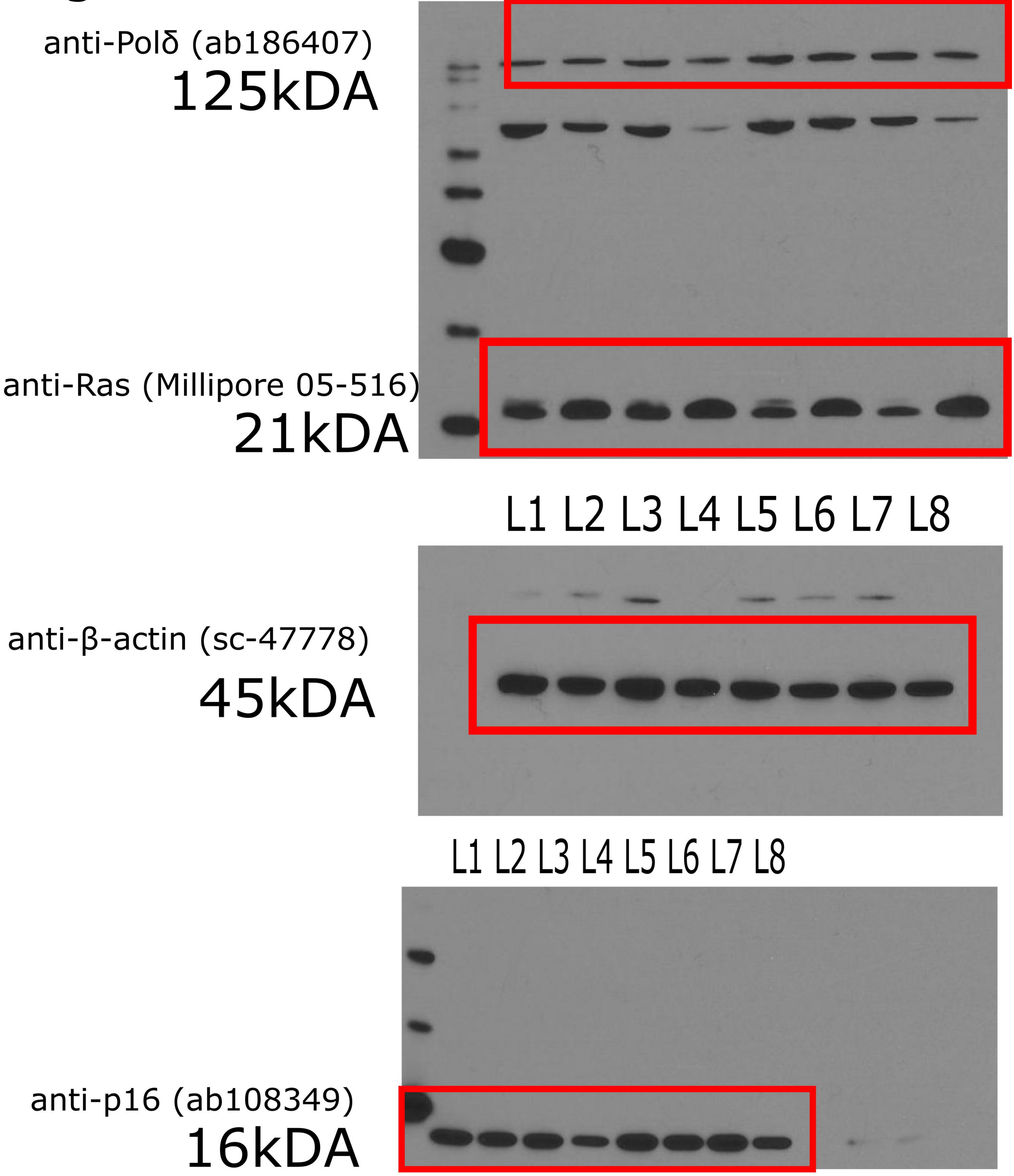

Lane 1: Day 2- SXPV cells infected with pbabe control
Lane 2: Day 2- SXPV cells infected with pbabe HRasG12V
Lane 3: Day 2- SXPV/Polη cells infected with pbabe control
Lane 4: Day 2- SXPV/Polη cells infected with pbabe HRasG12V
Lane 5: Day 8- SXPV cells infected with pbabe control
Lane 6: Day 8- SXPV cells infected with pbabe HRasG12V
Lane 7: Day 8- SXPV/Polη cells infected with pbabe control
Lane 8: Day 8- SXPV/Polη cells infected with pbabe HRasG12V

Fig7A

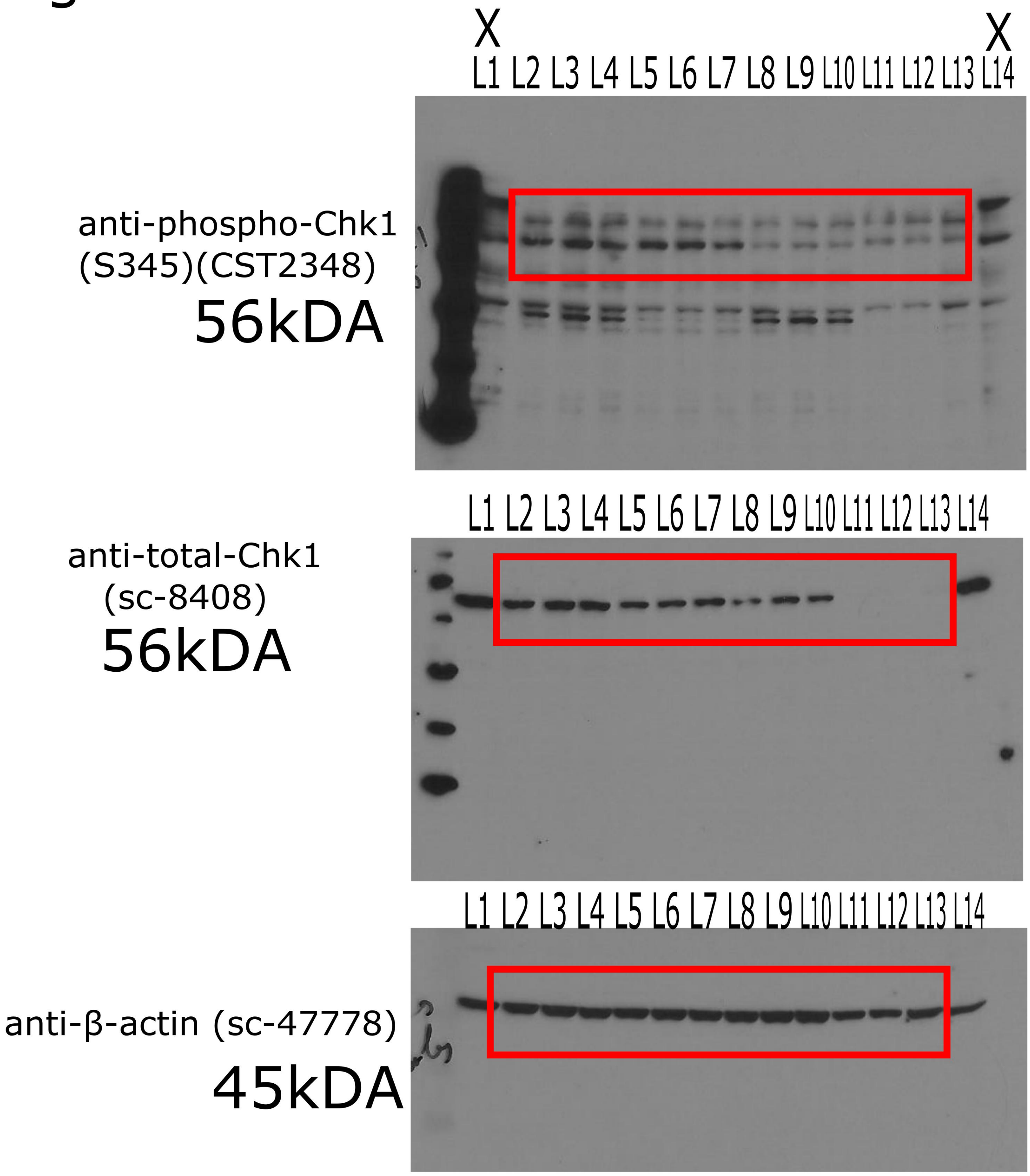

Lane 1: BJ5a cells treated with UV cells
Lane 2: Day 8- SXPV cells infected with pbabe control rep 1
Lane 3: Day 8- SXPV cells infected with pbabe control rep 2
Lane 4: Day 8- SXPV cells infected with pbabe control rep 3
Lane 5: Day 8- SXPV cells infected with pbabe HRasG12V rep 1
Lane 6: Day 8- SXPV cells infected with pbabe HRasG12V rep 2
Lane 7: Day 8- SXPV cells infected with pbabe HRasG12V rep 3
Lane 8: Day 8- SXPV/Polη cells infected with pbabe control rep 1
Lane 9: Day 8- SXPV/Polη cells infected with pbabe control rep 2
Lane 10: Day 8- SXPV/Polη cells infected with pbabe control rep 3
Lane 11: Day 8- SXPV/Polη cells infected with pbabe HRasG12V rep 1
Lane 12: Day 8- SXPV/Polη cells infected with pbabe HRasG12V rep 2
Lane 13: Day 8- SXPV/Polη cells infected with pbabe HRasG12V rep 3
Lane 14: BJ5a cells treated with aphidicolin

Fig7A continued

anti-phospho-Chk2
(Thr68)(CST13848)
70kDA

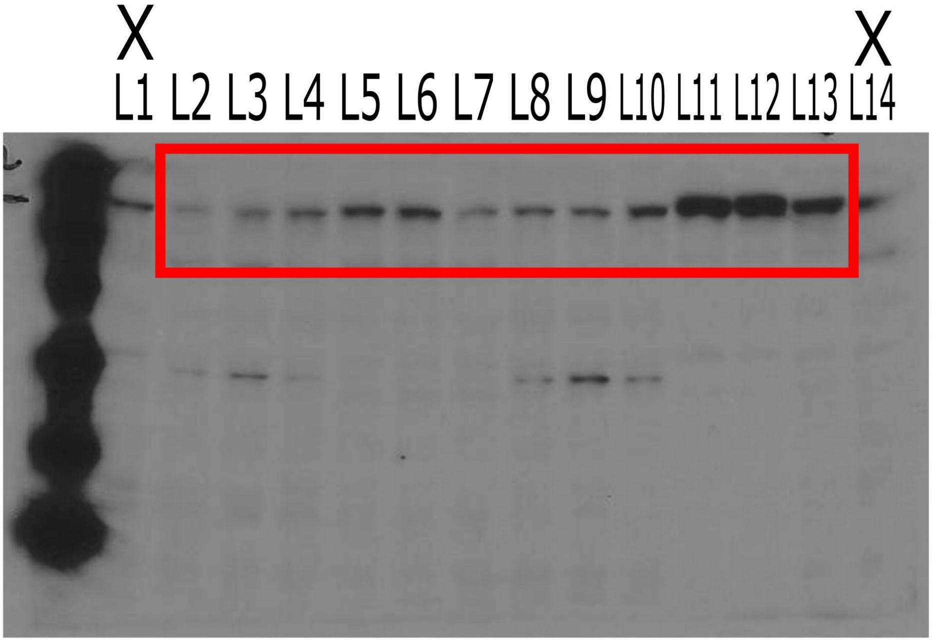

anti-total-Chk2
(CST13848)
70kDA

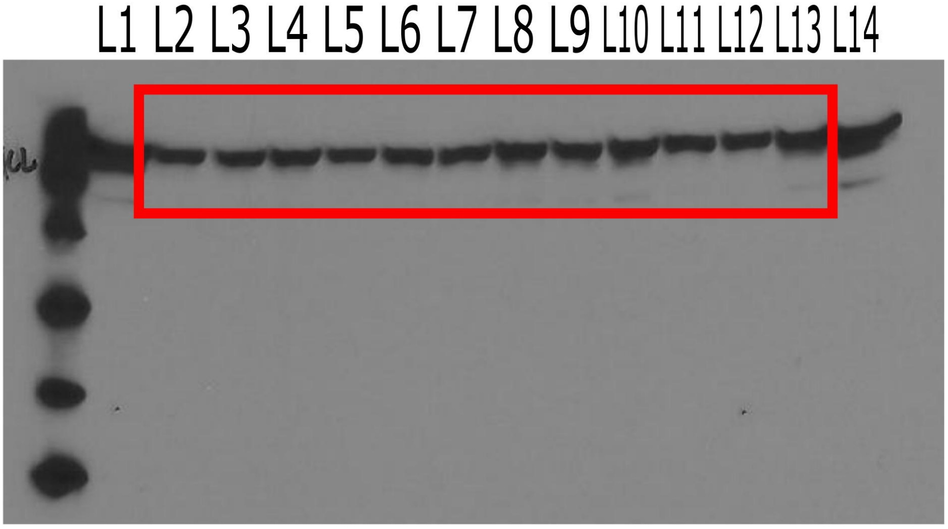

Lane 1: BJ5a cells treated with UV cells
Lane 2: Day 8- SXPV cells infected with pbabe control rep 1
Lane 3: Day 8- SXPV cells infected with pbabe control rep 2
Lane 4: Day 8- SXPV cells infected with pbabe control rep 3
Lane 5: Day 8- SXPV cells infected with pbabe HRasG12V rep 1
Lane 6: Day 8- SXPV cells infected with pbabe HRasG12V rep 2
Lane 7: Day 8- SXPV cells infected with pbabe HRasG12V rep 3
Lane 8: Day 8- SXPV/Polη cells infected with pbabe control rep 1
Lane 9: Day 8- SXPV/Polη cells infected with pbabe control rep 2
Lane 10: Day 8- SXPV/Polη cells infected with pbabe control rep 3
Lane 11: Day 8- SXPV/Polη cells infected with pbabe HRasG12V rep 1
Lane 12: Day 8- SXPV/Polη cells infected with pbabe HRasG12V rep 2
Lane 13: Day 8- SXPV/Polη cells infected with pbabe HRasG12V rep 3
Lane 14: BJ5a cells treated with aphidicolin

FigS1 rep 2

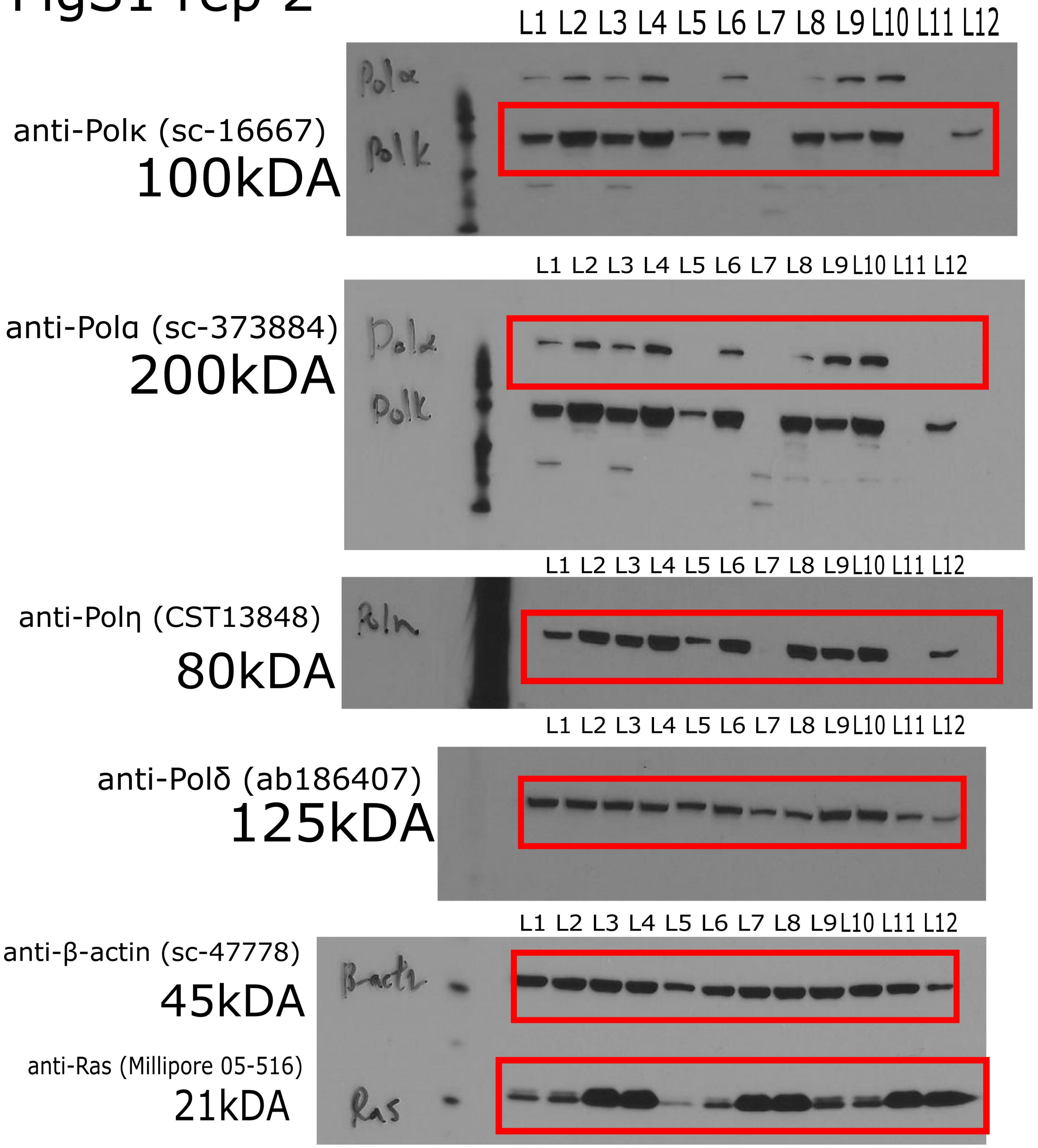

Lane 1: Day 2- BJ5a cells infected with pbabe control treated with DMSO rep 2
Lane 2: Day 2- BJ5a cells infected with pbabe control treated with MG132 rep 2
Lane 3: Day 2- BJ5a cells infected with pbabe HRasG12V treated with DMSO rep 2
Lane 4: Day 2- BJ5a cells infected with pbabe HRasG12V treated with MG132 rep 2
Lane 5: Day 4- BJ5a cells infected with pbabe control treated with DMSO rep 2
Lane 6: Day 4- BJ5a cells infected with pbabe control treated with MG132 rep 2
Lane 7: Day 4- BJ5a cells infected with pbabe HRasG12V treated with DMSO rep 2
Lane 8: Day 4- BJ5a cells infected with pbabe HRasG12V treated with MG132 rep 2
Lane 9: Day 8- BJ5a cells infected with pbabe control treated with DMSO rep 2
Lane 10: Day 8- BJ5a cells infected with pbabe control treated with MG132 rep 2
Lane 11: Day 8- BJ5a cells infected with pbabe HRasG12V treated with DMSO rep 2
Lane 12: Day 8- BJ5a cells infected with pbabe HRasG12V treated with MG132 rep 2

FigS1 rep 3

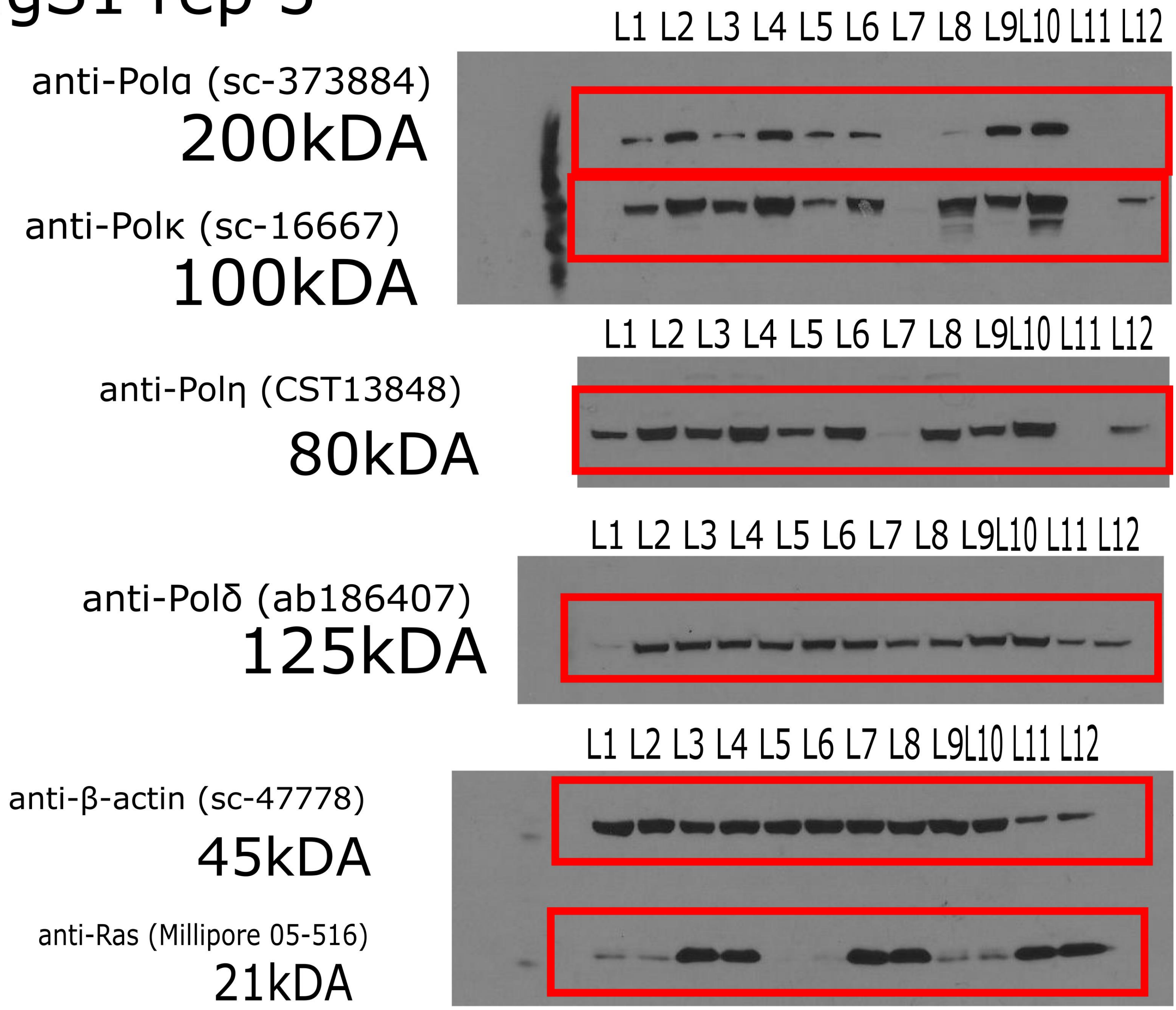

Lane 1: Day 2- BJ5a cells infected with pbabe control treated with DMSO rep 3
Lane 2: Day 2- BJ5a cells infected with pbabe control treated with MG132 rep 3
Lane 3: Day 2- BJ5a cells infected with pbabe HRasG12V treated with DMSO rep 3
Lane 4: Day 2- BJ5a cells infected with pbabe HRasG12V treated with MG132 rep 3
Lane 5: Day 4- BJ5a cells infected with pbabe control treated with DMSO rep 3
Lane 6: Day 4- BJ5a cells infected with pbabe control treated with MG132 rep 3
Lane 7: Day 4- BJ5a cells infected with pbabe HRasG12V treated with DMSO rep 3
Lane 8: Day 4- BJ5a cells infected with pbabe HRasG12V treated with MG132 rep 3
Lane 9: Day 8- BJ5a cells infected with pbabe control treated with DMSO rep 3
Lane 10: Day 8- BJ5a cells infected with pbabe control treated with MG132 rep 3
Lane 11: Day 8- BJ5a cells infected with pbabe HRasG12V treated with DMSO rep 3
Lane 12: Day 8- BJ5a cells infected with pbabe HRasG12V treated with MG132 rep 3
